# pkaPS: prediction of protein kinase A phosphorylation sites with the simplified kinase-substrate binding model

**DOI:** 10.1186/1745-6150-2-1

**Published:** 2007-01-12

**Authors:** Georg Neuberger, Georg Schneider, Frank Eisenhaber

**Affiliations:** 1 IMP – Research Institute of Molecular Pathology, Dr. Bohr-Gasse 7, A-1030 Vienna, Austria

## Abstract

**Background:**

Protein kinase A (cAMP-dependent kinase, PKA) is a serine/threonine kinase, for which ca. 150 substrate proteins are known. Based on a refinement of the recognition motif using the available experimental data, we wished to apply the simplified substrate protein binding model for accurate prediction of PKA phosphorylation sites, an approach that was previously successful for the prediction of lipid posttranslational modifications and of the PTS1 peroxisomal translocation signal.

**Results:**

Approximately 20 sequence positions flanking the phosphorylated residue on both sides have been found to be restricted in their sequence variability (region -18...+23 with the site at position 0). The conserved physical pattern can be rationalized in terms of a qualitative binding model with the catalytic cleft of the protein kinase A. Positions -6...+4 surrounding the phosphorylation site are influenced by direct interaction with the kinase in a varying degree. This sequence stretch is embedded in an intrinsically disordered region composed preferentially of hydrophilic residues with flexible backbone and small side chain. This knowledge has been incorporated into a simplified analytical model of productive binding of substrate proteins with PKA.

**Conclusion:**

The scoring function of the pkaPS predictor can confidently discriminate PKA phosphorylation sites from serines/threonines with non-permissive sequence environments (sensitivity of ~96% at a specificity of ~94%). The tool "pkaPS" has been applied on the whole human proteome. Among new predicted PKA targets, there are entirely uncharacterized protein groups as well as apparently well-known families such as those of the ribosomal proteins L21e, L22 and L6.

**Availability:**

The supplementary data as well as the prediction tool as WWW server are available at .

**Reviewers:**

Erik van Nimwegen (Biozentrum, University of Basel, Switzerland), Sandor Pongor (International Centre for Genetic Engineering and Biotechnology, Trieste, Italy), Igor Zhulin (University of Tennessee, Oak Ridge National Laboratory, USA).

## Open peer review

This article was reviewed by Erik van Nimwegen (Biozentrum, University of Basel, Switzerland), Sandor Pongor (International Centre for Genetic Engineering and Biotechnology, Trieste, Italy) and Igor Zhulin (University of Tennessee, Oak Ridge National Laboratory, USA). For the full reviews, please go to the Reviewers' comments section.

## Background

Phosphorylation is one of the biologically most important post-translational modifications known today. Eukaryote kinases, which are the enzymes that are responsible for this type of chemical alteration, transfer phosphate moieties onto the hydroxyl groups of serines, threonines or tyrosines of substrate peptides. Phosphorylation plays a key role in a large set of signal transduction pathways and is known to regulate the functions of a vast number of different proteins. Not only are substrate motifs for phosphorylation found in proteins from various cellular contexts, there are also >500 kinases [1] with at least partly non-overlapping substrate specificities encoded in each of the higher eukaryote genomes. This broad distribution, coupled with the potential medical applications, makes them interesting research targets with regard to their role in signaling cascades. Therefore, it is important to determine the complete protein substrate set for each kinase. The sheer number of yet uncharacterized proteins implies that a lot of phosphorylation motifs remained undetected so far. Accurate *in silico *predictors recognizing kinase substrates from their amino acid sequences are desirable to bring this task closer to a solution. A low false-positive prediction rate is especially important in this context.

Protein kinase A (PKA), alternatively called cAMP-dependent protein kinase, is one of the best studied members of the kinase group of enzymes and, therefore, appears among the most attractive targets for substrate site predictor development. It is actually the first kinase for which the crystal structure has been resolved [2,3]. PKA acts on serine and, to a lesser extent, threonine residues that are embedded in a specific recognition motif. In its first characterizations, the PKA motif was described as consisting of arginines at the 3^rd ^and 2^nd ^positions prior to the phosphorylation site, and of a large hydrophobic amino acid immediately thereafter [4-7].

Several groups already applied various approaches for predicting PKA phosphorylation sites from primary protein sequence. NETPHOS [8] was one of the first to outperform simpler PROSITE-like approaches [9-11] by applying artificial neural networks. A more recent version, NETPHOSK [12], makes kinase-specific predictions. SCANSITE 2.0 uses position-specific scoring matrices (PSSM) to predict phosphorylation motifs for 62 different kinases, again including PKA [13]. PREDPHOSPHO is a kinase-specific predictor that uses support vector machines [14]. GPS does not use standard machine learning approaches but implements a so-called group-based scoring technique, which makes use of the BLOSUM62 matrix to score distances between query sequences and known clusters of kinase substrate peptides [15,16]. As GPS focuses on straight sequence similarity traits, the likelihood for GPS to recognize query peptides as phosphorylation targets that are similar to known sites is especially high whereas GPS might have difficulties if it is confronted with unusual substrate examples of the same kinase that are not reflected in the learning set. Among these tools, GPS [15,16] and PREDPHOSPHO [14] appear to have highest accuracy. Although the sequence sets used for testing are limited, their sensitivities are clearly below 90% for specificities estimated to be close to 90%. As more than 10% of the query sites are expected to be misclassified, database-wide studies that rely solely on current predictors cannot produce reliable results.

In order to achieve higher sensitivity and specificity, major improvements are needed. In this work, we implemented two new aspects: (i) Since there is no "average phosphorylation site", high prediction accuracy can only be achieved if the function for scoring of putative phosphorylation sites is specific for each kinase system. In our approach, the scoring function is thought to estimate the probability of productive binding of the respective substrate protein segment with the binding site of PKA; thus, the scoring function is a simplified physical model of the binding process [17-22]. (ii) The motif regions that are used to discriminate between true sites and non-permissive targets should be as long as possible. These shall include all substrate sequence stretches that influence the binding process and should not be restricted to the region of the motif that is most conserved in terms of amino acid types. It is also necessary to consider properties of correlated motif positions [23].

It should be noted that, for most post-translational modifications, only a handful of substrate proteins per modifying enzyme is known. Even for the better studied cases, the available experimental information can only reliably parameterize a scoring function with a small number of fitted values. In similar cases of predictor development such as for GPI lipid anchoring [17], N-terminal N-myristoylation [20], prenylation [21] and peroxisomal targeting [22,24], our simplified substrate protein binding model has been successfully applied. It should be noted that, in all these cases, the sequence signal coding for the posttranslational modification or the translocation is located either at the N- or C-terminal end of the polypeptide chain. In this work, we wanted to test the approach for an internal sequence signal.

PKA-dependent phosphorylation is an excellent example in this context since the rich experimental data allow for the derivation of a quite accurate qualitative binding site model as we show in this work. Not only are there more than 200 documented phosphorylation sites for PKA. The available sequence data is also accompanied by other valuable heterogeneous information such as 3D data and mutation experiments [3,25].

## Results

### Overview

The whole work consist of two major parts – first, the derivation of the property pattern that characterizes sequence segments with PKA phosphorylation sites and, second, the development and the validation of a prediction tool for the recognition of PKA phosphorylation sites in query sequences.

The following four sections of the Results ("The motif length", "Positive charge in the N-terminal flank", "Polarity and flexibility in the C-terminal flank", "Phylogenetic variation of the substrate binding site of PKA") are dedicated to the derivation of the sequence motif coding for PKA phosphorylation sites. This work is based on analyses of the sequence environment of known phosphorylation sites in substrate proteins and of the PKA sequences and structures. We correlate amino acid compositions at various alignment positions with physical properties of amino acid residues. As major results, we obtain the sequence length of the motif and the pattern of physical properties in various sequence segments surrounding the phosphorylation site. Moreover, if several phosphorylation sites occur in one protein, they tend to be sequentially clustered.

The next three sections ("Predictor description and the self-consistency test", "Neighbor-jackknife test", "Summary of the prediction performance and comparison to other tools") describe the development of the prediction tool and its validation with the self-consistency test and a rigorous cross-validation procedure called neighbor-jackknife test (exclusion of groups of sequentially similar proteins). The specificity and the sensitivity values are close to 95% and, thus, superior compared with previously published predictors.

The succeding section of the Results ("Prediction of PKA targets within the human proteome") describes the application of the predictor to the human proteome. Among new predicted PKA targets, there are entirely uncharacterized protein groups as well as apparently well-known families such as those of the ribosomal proteins L21e, L22 and L6. The last section of the Results ("Description of the associated WWW site") supplies information about the PKA WWW server.

#### The motif length

The deduction of accurate motif boundaries is not straightforward, as this region also comprises positions that make only minor contributions to substrate recognition by PKA. For example, these include residues that interact only weakly with the receptor or which are context-dependent upon neighboring positions. As a consequence, it is helpful to base such estimations on a standard model which has already been successfully applied in related situations.

The concept of a linker-embedded binding motif is utterly suited for this task. The underlying assumption is that the peptide stretch which binds to the receptor enzyme and which is buried in the catalytic cleft must first be made accessible for interaction: as part of an intrinsically disordered region, through a permanent native location on the surface of the globular part of the substrate protein or via exposure after an induced conformational change. As a consequence, the flanking regions which connect the sequence segment that fits into the catalytic cavity and the rest of the substrate protein must have sufficient conformational flexibility and hydrophilicity. Such a motif structure has already been observed and successfully applied in predictor development [21-24,26]. Recent work by Dunker and co-workers further confirms the applicability of this model to protein phosphorylation motifs as they find evidence for inherently disordered regions surrounding phosphorylated residues. They used a similar formulation of the concept for "disorder enhanced" prediction of phosphorylation sites [27].

**Figure 1:**
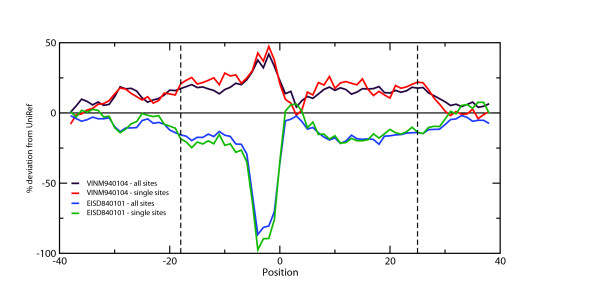
**Variation of hydrophobicity and of flexibility over the motif region**. The graph depicts the mean value deviations of the hydrophobicity-related property EISD840101 [29] and the flexibility scale VINM940104 [30] over the 81 positions that encompass the learning set sites. The mean values are presented as deviations from the UNIREF average (baseline) in percent of UNIREF standard deviations. The plots were smoothed by applying sliding windows (running averages) over 5 residues. Mean values were calculated using two different sequence sets: (i) one that contains all entries from the learning set, and (ii) one that consists of all proteins that are phosphorylated only once in the learning set. The difference between these two curves is not dramatic although, as a trend, the property values appear to fall back more sharply to the database values if only proteins with single PKA phosphorylation sites are taken into account.

Mean values of amino acid property indices [23,28] (including many flexibility and hydrophobicity scales) were calculated over a gapless multiple alignment of learning set sequences which consists of the modified sites in the center together with 40 flanking residues on each side. Sequence redundancy was removed by applying a method which involves frequencies of identical residues on alignment positions -6 to +6 (Materials and Methods). Exemplarily, we show the outcomes obtained for the hydrophobicity scale EISD840101 [29] and for VINM940104 [30] as flexibility measure in Figure 1.

The calculated values deviate from the database averages over a sequence stretch that covers about twenty positions both the N- and at the C-terminal side of the documented phosphorylation site. The curves fall back to the average database values with increasing distance from the phosphorylated site. Moreover, similar behavior is exhibited by many other hydrophobicity- and flexibility-related properties (data not shown). It appears also interesting that this region is slightly longer on the C-terminal side than on the N-terminal one. This might be a result of the more hydrophobic nature of the residues that lie adjacent to the phosphorylated site on the C-terminal side. As depicted in Figure 1, the motif boundaries cannot be boiled down unambiguously to a unique position. We set the edges well into the regions where the property mean values do not fall below the steady level of approximately ± 20%. The resulting region is defined from positions -18 to +23 and, thus, we estimate the total length of the sequence signal for PKA-dependent phosphorylation as 42 positions.

**Figure 2:**
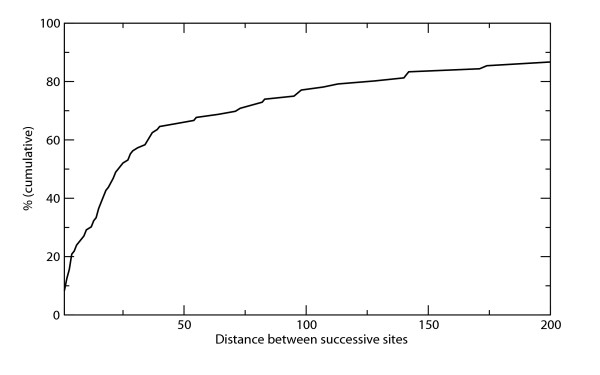
**Cumulative distribution of distances between successive sites in learning set proteins with multiple phosphorylated serine/threonine residues**. The figure demonstrates that about two thirds of all distances are within the extended motif length of approximately 50 positions. The maximum distance, which exceeds the displayed x-axis, is 1759 amino acids.

The significance of multiple phosphorylated residues within the same motif region is an important issue that needs to be addressed (see also legend to Figure 1). We find that pairs of phosphorylated serines/threonines are not separated farther than the 50 residues in the sequence in two thirds of all cases (Figure 2). This threshold is just about the motif length derived above. Theoretically, every proximal neighboring site would prolong one of the linkers by at least the distance between the sites. From a biological point of view, it appears reasonable to pack multiple phosphorylation sites closely together. In such a situation, long additional linker stretches that would be necessary for maintaining an inherent structural disorder in the environment of phosphorylation sites [27] are avoided.

#### Positive charge in the N-terminal flank

Historically, charge requirements were the first observed characteristics of the PKA motif. Kinetic studies at the end of the 1970s revealed a cluster of positive residues directly N-terminally of the phosphorylated site as main determinant for PKA substrate specificity. The main constituents of this cluster are the 2^nd ^and 3^rd ^positions prior to the phosphorylated serine or threonine. Kemp *et al*. [5] postulated that at least one arginine should be present at one of these locations. Moreover, replacement of the arginine by lysine was reported to cause less activity loss than substitutions by other amino acids. In another study [31], the adjacent arginines were positioned at various distances from the phosphorylated site and activity measurements were performed. The results demonstrated that the binding affinity is indeed highest at positions -3/-2 and decreases with increasing distance from the site.

**Figure 3:**
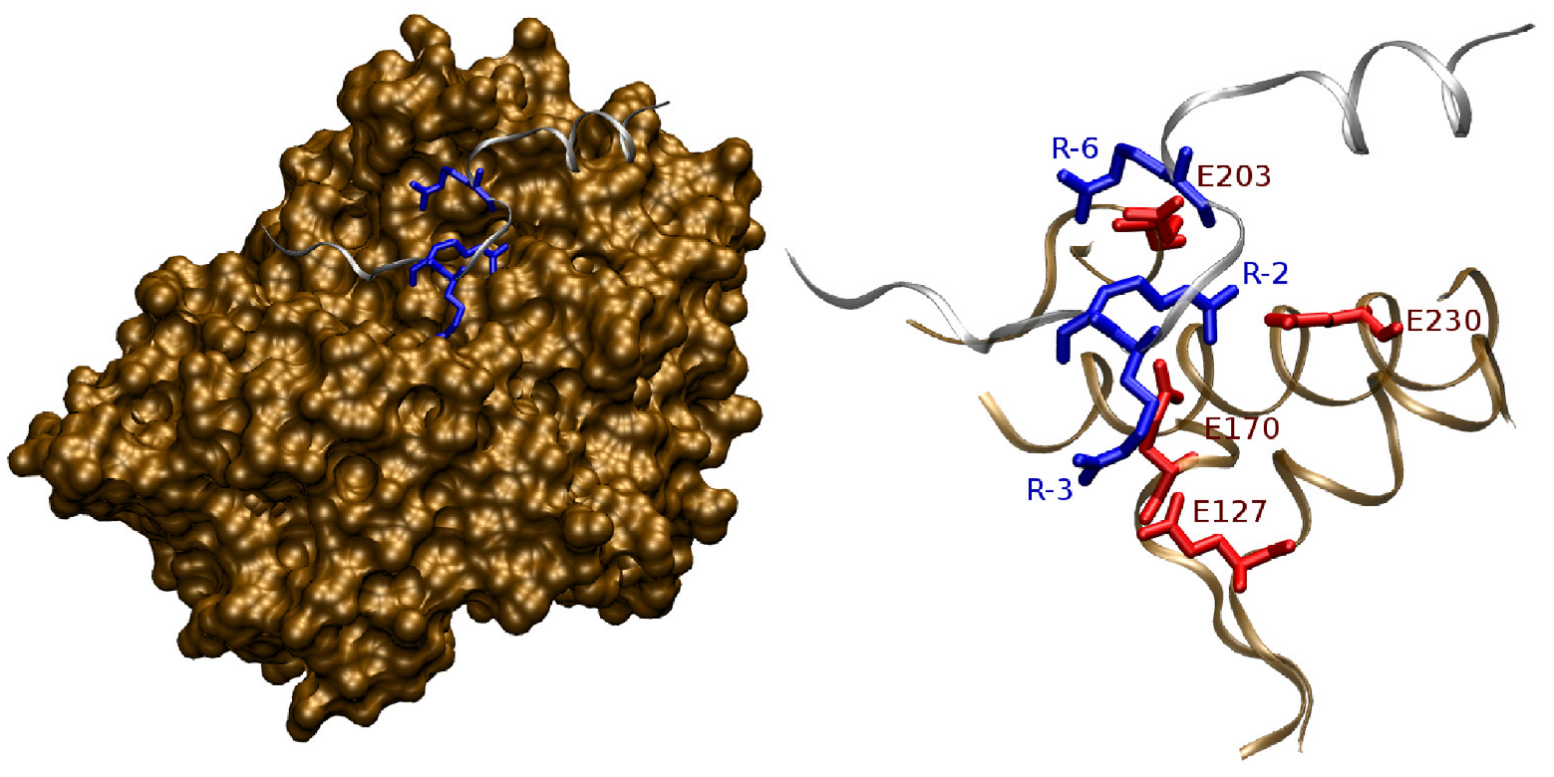
**Structure of the inhibitor peptide PKI bound to the PKA enzyme: N-terminal region of the substrate**. Key arginines from the substrate peptide (RCSB Protein Data Bank entry 1JLU [92]) are highlighted. The left part of the figure shows the surface of PKA in ochre, the backbone of the substrate peptide in silver and the arginines -6, -3 and -2 of the substrate in blue. Arginines -3 and -2 interact with the binding cleft and thereby make major contributions to substrate specificity. A set of acidic enzyme residues interacts with these arginines (zoomed detail-view to the right): Glu170 and Glu230 for Arg-2, Glu127 for Arg-3 and Glu203 for Arg-6 [3]. The pictures were generated using VMD [93].

The requirement for positively charged residues is depicted in the 3D structure of PKA bound to an inhibitor peptide (Figure 3). The arginines at positions -3 and -2 interact with Glu127 and Glu170/Glu230 of the PKA enzyme, respectively [3]. Both substrate residues make close contacts with the enzyme in a spatially restricted binding pocket, explaining their importance in determining substrate specificity. In this structure, the arginine at position -6 also contributes to substrate recognition by interacting with Glu203.

**Figure 4:**
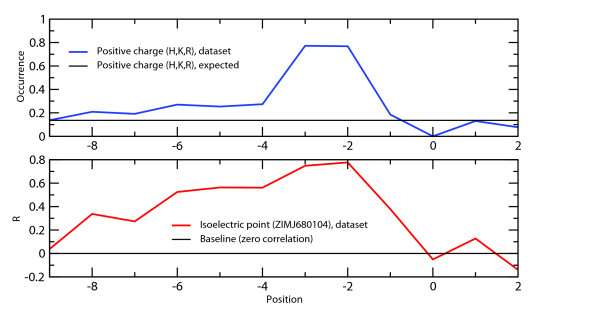
**Preference for positive charge at positions located N-terminally with regard to the phosphorylated site**. The upper graph depicts the increased occurrence of positively charged residues (His, Lys, Arg) compared to the expected database occurrence of 13.6% (deduced from UNIREF). The lower part of the figure shows the correlation coefficients R between amino acid frequencies and ZIMJ680104 (isoelectric point) [87] property values. Both plots demonstrate that the preference for basic residues is highest at positions -3 and -2, but encompasses at least the entire region between amino acids -6 and -2.

The requirement for positive charge is highest for residues -2 and -3 but can be detected as far as 6 to 8 residues prior to the phosphorylated serine (Figure 4). Several studies focus on the role of position -6 as this residue apparently interacts with the PKA enzyme [3,7]. In contrast, it is unclear how the amino acids at positions -5 and -4 contribute to substrate specificity. Although positive charge at these locations is as much favored as for position -6, neither of them makes close contacts with PKA in any solved structure. The reason could lie in a variable structural context of this N-terminal region. The currently resolved substrate-bound 3D structures have typically been obtained using the same inhibitor peptide (PKI). Thus, other, yet unknown conformations might exist if the bound peptide does not involve a positively charged residue at position -6. Alternatively, long-range charge interactions might contribute to substrate specificity at these positions. The preference for positive charge is further confirmed by Songyang *et al*. [25], who used an oriented peptide library to demonstrate that the positional range -4 to -1 has strong preferences for arginine and to, a lesser extent, for histidine or lysine.

Physico-chemical preferences in the region prior to the phosphorylation site are complemented with flexibility and polarity requirements, e.g. for the property VINM940103 (normalized flexibility parameters [30], R ≥ 0.62) at positions -8 to -6 and -4, or for the hydrophilicity-related scales EISD840101 [29] (R ≥ -0.66 at position -3 and -0.69 at position -2) and KRIW790102 [32] (R = 0.60 at positions -7, -6 and -4). Although these might be a remnant of charge requirements, it seems clear that a substitution of arginine by hydrophilic residues is less disfavored than an exchange by bulky, apolar amino acids.

#### Polarity and flexibility in the C-terminal flank

**Figure 5:**
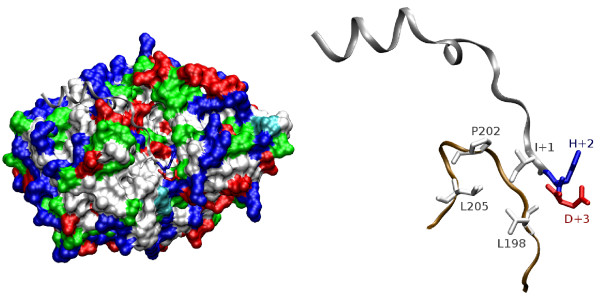
**Structure of the inhibitor peptide PKI bound to the PKA enzyme: C-terminal region of the substrate**. Overall (left) and detail views (right) of the substrate region that lies on the C-terminal side of the phosphorylated serine in complex with the kinase PKA (RCSB Protein Data Bank entry 1JLU [92]) are shown. Ile+1, His+2 and Asp+3 of the PKI substrate as well as the surface of the PKA enzyme to the left are colored according to residue types: white/gray for apolar, green for polar, blue for basic, and red for acidic amino acids. Compared with Figure 3, the orientation of the complex roughly corresponds to a counterclockwise rotation of 90 degrees around the vertical axis. The detail view to the right shows the hydrophobic patch at the surface of PKA which interacts with the substrate residue that lies C-terminally adjacent to the phosphorylated site. The pictures were generated using VMD [93].

The residue at position +1 lies in vicinity of a hydrophobic pocket that is built up by the side chains of Leu198, Pro202 and Leu205 (Figure 5). As a consequence, a large hydrophobic residue was found to be favored at this substrate position [7]. A value of R = 0.78 for NAKH900109 (amino acid composition of membrane proteins [33]) confirms the detected tendency for hydrophobic, apolar residues. Also, analysis of mean value deviations from the expected database average indicates a preference for amino acids that occur more frequently in β-strands. Properties such as GEIM800105 (β-strand indices [34]) or KANM800104 (average probability for inner β-sheet [35]) produce significant t-values of 2.71 (99.2%) and 2.59 (98.9%), respectively. These secondary structure scales typically have elevated property values for aliphatic and aromatic amino acids.

Interestingly, correlation effects can be detected between positions +1 and +4, as indicated by an F-value of 1.38 (96.6%) for the property GEIM800107 (β-strand indices for β-proteins [34]). Few data about the role of residue +4 is available from the literature, as this position is missing in the currently resolved 3D structures. It has no clear amino acid preferences, although it is preferentially less polar than the clearly hydrophilic surrounding positions (data not shown). Its spatial location in vicinity of the hydrophobic patch (Figure 5) combined with the correlations with residue +1 could suggest that positions +1 and +4 both may interact with the apolar surface loop of PKA. However, alternative conformations which involve an apolar residue at position +3 also appear possible.

The intermediary positions +2 and +3 can be characterized by a preference for small residues. Numerous size-related scales such as FASG760101 [36] (R of -0.62 and -0.63 for positions +2 and +3, respectively) produce significant correlation coefficients. To some extent, position +3 also seems to favor flexible amino acids, as indicated by a correlation coefficient R of 0.65 for VINM940102 [30]. The respective substrate positions indeed lie in a spatially constrained region at the mouth of the binding cavity (Figure 5), which explains the appearance of size restrictions.

#### Phylogenetic variation of the substrate binding site of PKA

**Figure 6:**
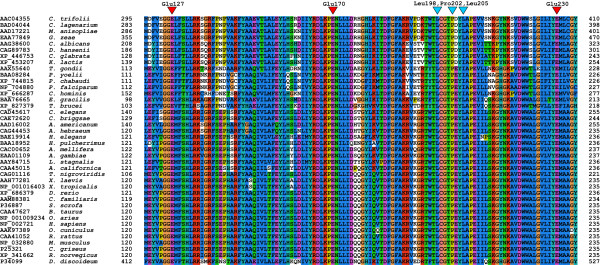
**Multiple alignment of the binding site regions across PKA orthologue sequences**. Starting with the mouse sequence (accession NP_032880) of the protein in the crystal structure 1JLU [92], we searched for orthologues of the catalytic subunit of PKA with the ANNOTATOR suite [45]. In the alignment (generated with T-COFFEE [94]), we present 40 variants thereof ranging as far as from yeast to human (sequence position numbering is without leading methionines according to the 1.29 Å rule [56,57]). The figure focuses on the protein polypeptide stretch that encompasses the residues forming the surface of the binding site at substrate position from -3 to +1. Red triangles (at Glu127, Glu170 and Glu230 in the numbering of 1JLU without the leading methionine in NP_032880) mark positions that form the pocket for substrate residues -3 and -2. Blue triangles (at Leu198, Pro202 and Leu205) mark the hydrophobic pocket-forming positions that accept substrate residue +1 [4–7].

When collecting the learning set substrate proteins, we found 50% human and 89% mammalian example sites. The remaining 11% were from other metazoan species, yeasts and plants (see Materials and Methods for detail). We wished to estimate to which extent substrates and enzymes from various organisms are exchangeable with respect to PKA-dependent phosphorylation. In Figure 6, we show the alignment of the sequences of the catalytic subunit of PKA in a large variety of organisms spreading from yeast to human. Positions that are critically important for binding the substrate protein stretch are marked with triangles. Not only are these positions 100% conserved among all sequences shown, but even their sequence environment is almost unchanged among taxa. Therefore, we suggest that substrates for the human PKA are most likely also substrates for PKA of other taxa and a predictor for recognizing human substrates can also be used for finding PKA substrates in other eukaryote organisms.

#### Predictor description and the self-consistency test

The motif structure that was presented in the preceding sections served as a basis for the generation of a prediction tool. The final version of the predictor, called "pkaPS", uses one profile over 13 sequence positions and 14 physico-chemical property terms. In the self-consistency test, the pkaPS predictor generates scores S ≥	 0 for 236 out of 239 (98.7%) positive examples from the learning set, and, thus, correctly predicts these sequences as potential substrates for PKA-dependent phosphorylation.

The three entries that are not predicted are summarized in Table 1. Although all of them produce profile scores S_profile _above zero, the three database sites (i) Ser10 from the rat brain myelin basic protein [37], (ii) Ser356 from the rat liver fructose-1,6-bisphosphatase [38] and (iii) Ser197 from human cyclin C1 [39] obviously differ from the consensus represented by the scoring function to a considerable extent. Among other unmet requirements, the charge pattern on the N-terminal side of the reported phosphorylation sites is deviant. Typically, positive charged residues are observed and negative charges are absent. Actually, none of these sites harbors an arginine at either of the important positions -3 or -2. Therefore and given the current knowledge on substrate binding, it is difficult to imagine how these targets fit into the binding site of PKA. Since experimental protocols for determining phosphorylation sites are non-trivial and the reports are of considerable age, an experimental re-examination these cases would be advisable.

**Table 1:** Results of the self-consistency test

Score	Profile	*T*_*j*_	Access.	Site	Sequence	Reference
-0.381	1.114	*T*_*1*_,*T*_*2*_	P02687	10	---------AAQKRPSQRSKYLASASTMDHARHGFLPRHRDT	Kishimoto *et al*. 1985 [37]
-1.338	0.495	*T*_*1*_-*T*_*4*_	P19112	356	SRPSLPLPQSRARESPVHSICDELF-----------------	Ekdahl 1987 [38]
-1.368	0.069	many	P24385	197	RKHAQTFVALCATDVKFISNPPSMVAAGSVVAAVQGLNLRSP	Sewing & Müller 1994 [39]

Phosphorylated sites from the learning set that are not predicted by the normally parameterized pkaPS predictor (false-negatives in the self-consistency test). The listed penalties *T*_*j *_are the terms which make the highest contributions to the negative overall scores.

The expected rate of false-positive predictions can directly be estimated using the set of 1026 negative examples. For a given serine or threonine residue of a query sequence, the probability of true-negative prediction lies at 93.5% (F_p_-rate of 6.5%). This set was used to generate an empirical score distribution of negative examples. In order to obtain a value for the false-positive rate for any generated score S, an analytical approximation of this score distribution was determined (Materials and Methods).

#### Neighbor-jackknife test

Thorough cross-validation tests are needed in order to assess whether the score function is stably parameterized by the learning set. The pkaPS tool was subjected to a strict cross-validation test where the query sequence in addition to sequences which share more than 30% of identical amino acids with the query were excluded from the parameterization procedure (neighbor-jackknife test, Materials and Methods).

As summarized in Table 2, 10 out of the 239 (4.2%) sites from the learning set were not predicted by pkaPS. As expected, the entries that were not predicted in the self-consistency test were not recognized in the cross-validation test either. In this test, two entries (Q13002, position 697 and P24385, position 197) had profile scores below zero. Therefore, we think that the learning set is still a little bit too small to stably determine the profile.

**Table 2:** Results of the neighbor-jackknife test

Score	Profile	T_j_	Access.	Site	Sequence	Reference
-0.108	0.268	*T*_*7*_,*T*_*8*_	P02687	33	SASTMDHARHGFLPRHRDTGILDSLGRFFGSDRGAPKRGSGK	Kishimoto *et al*. 1985 [37]
-0.158	0.733	*T*_*2*_,*T*_*4*_	P12336	489	VLVFTLFTFFKVPETKGKSFDEIAAEFRKKSGSAPPRKATVQ	Thorens *et al*. 1996 [61]
-0.174	0.560	*T*_*4*_,*T*_*7*_	P24155	643	RFKQEGVLSPKVGMDYRTSILRPGGSEDASTMLKQFLGRDPK	Tullai *et al*. 2000 [69]
-0.264	0.145	*T*_*2*_	P02643	19	GDEEKRNRAITARRQHLKSVMLQIAATELEKEEGRREAEKQN	Huang *et al*. 1974 [82]
-0.367	0.113	*T*_*2*_,*T*_*10*_	Q07954	4517	PTNFTNPVYATLYMGGHGSRHSLASTDEKRELLGRGPEDEIG	Li *et al*. 2001 [83]
-0.469	0.564	*T*_*4*_,*T*_*12*_	P25961	473	VAIIYCFCNGEVQAEIRKSWSRWTLALDFKRKARSGSSSYSY	Blind *et al*. 1996 [62]
-0.469	-0.028	*T*_*2*_,*T*_*12*_	Q13002	697	KIEYGAVEDGATMTFFKKSKISTYDKMWAFMSSRRQSVLVKS	Wang *et al*. 1993 [84]
-0.619	0.891	*T*_*1*_,*T*_*2*_	P02687	10	---------AAQKRPSQRSKYLASASTMDHARHGFLPRHRDT	Kishimoto *et al*. 1985 [37]
-1.813	0.038	*T*_*1*_-*T*_*4*_	P19112	356	SRPSLPLPQSRARESPVHSICDELF-----------------	Ekdahl 1987 [38]
-1.867	-0.409	many	P24385	197	RKHAQTFVALCATDVKFISNPPSMVAAGSVVAAVQGLNLRSP	Sewing & Müller 1994 [39]

Phosphorylated sites from the learning set that are not predicted by pkaPS in the neighbor-jackknife test. The three entries with the lowest scores are not predicted in the self-consistency test either (Table 1). The listed penalties *T*_*j *_are the terms which make the highest contributions to the negative overall scores.

All of the seven entries that were predicted in the self-consistency test but not in the neighbor-jackknife test have only marginally negative scores between zero and -0.5. Only the three entries that had scores below zero in the self-consistency test also had an S < -0.5 in the neighbor-jackknife test (see Tables 1 and 2). We think that the score interval between 0.0 and -0.5 represents a twilight zone.

#### Summary of the prediction performance and comparison to other tools

To conclude, the prediction performance of the pkaPS tool is considerable. Its sensitivity lies in the range of at least 95.8% as estimated from the neighbor jackknife test, and is as high as 98.7% in the self-consistency test. At the same time, a specificity of 93.5% could be achieved.

The pkaPS tool was compared to a set of currently available phosphorylation predictors (Table 3). Unfortunately, a comparison of these tools on the same test set was impossible due to the lack of publicly available untrained versions of the tools that could be used for cross-validation tests. Hence, the comparisons were based on sensitivity and specificity values which were taken from the original papers. The performance values from older publications do not straightforwardly compare with those in this work. In general, prediction methods can be expected to show decreased performance when tested on enlarged, more recent and diverse sequence sets. More importantly, the accuracy measured for a prediction tool is also influenced by the rigor of the cross-validation test. This type of test should determine how predictors perform on query sequences that are dissimilar to the learning set examples. In our work, a strict method has been applied, the neighbor-jackknife test. In the leave-one-out procedure, we excluded not only the entry under consideration but also all sequentially similar examples.

**Table 3:** Prediction performances of available algorithms compared to pkaPS.

	Prediction performance		
		
Algorithm	*S*_*n *_[%]	*S*_*p *_[%]	Reference
DISPHOS	ca. 76	ca. 85	Iakoucheva *et al*. 2004 [27]
SCANSITE	70.7	92.9	Zhou *et al*. 2004 [15]
NETPHOSK	79	89	Blom *et al*. 2004 [12]
GPS	88.9	90.6	Xue *et al*. 2005 [16]
PREDPHOSPHO	88.3	91.1	Kim *et al*. 2004 [14]
pkaPS	95.8	93.5	this work

The table shows the prediction performances of five other programs compared to the worst performance (*S*_*n *_from neighbor jackknife-test) of the pkaPS predictor. All listed values except for those from DISPHOS refer to PKA-specific versions of the prediction tools. The pkaPS program outperforms all currently available methods that can be used to detect PKA-dependent phosphorylation. The sensitivities (*S*_*n*_) and specificities (*S*_*p*_) were directly taken from the original papers. Iakoucheva *et al*. [27] provide two possible values for the specificity as performance measure for the DISPHOS predictor, one that takes into account the possible occurrence of noise in the negative learning set (higher *S*_*p*_) and one that does not (lower *S*_*p*_). For DISPHOS, the *S*_*n *_value of 76% for serine (none is mentioned for threonine) and the higher, estimated specificity value of 85% (for serine) were used. The prediction performance for the SCANSITE program was not taken from the corresponding publication (Obenauer *et al*. 2003 [13]) but from Table 2 in the GPS paper [15]. The reason is that no evaluation of the SCANSITE performance in detecting sites for phosphorylation by PKA could be found in the paper from Obenauer et al. 2003 [13]. Here, the values for the low stringency cut-off were taken.

As judged from the published predictor performance ratings, pkaPS provides a better specificity and sensitivity than all other currently available tools. Among these methods, only DISPHOS [27] is a predictor for "average phosphorylation" sites without considering kinase specificity. All other tools have implementations for specific kinases including PKA. The algorithms that come closest to the performance of pkaPS are PREDPHOSPHO, which uses a support vector machine based implementation [14], and GPS, which rests upon a group-based scoring method [15].

#### Prediction of PKA targets within the human proteome

In addition to thorough cross-validation tests, the performance of the pkaPS tool was studied by analyzing predicted PKA-dependent phosphorylation sites in the human proteome. The human protein sequences were retrieved from the NCBI FTP-site (40877 sequences, September 14^th ^2006 at [40]). From a total of 2,485,866 serines and threonines, 258,271 (10.4%) were predicted as putative phosphorylation sites with scores *S *> 0. In our understanding, the list of predicted sites contains (a) true phosphorylation PKA sites, (b) sites that are phosphorylated *in vitro *by PKA but not *in vivo *due to the absence of biological context (see the comment on hidden signals in the discussion and in ref. [41]) and (c) real false-positive predictions of phosphorylated serines/threonines that are in sequence stretches without capability of productive interaction with the catalytic site of PKA. The consideration of additional functional sequence regions has a significant impact on the rate of predicted PKA-dependent phosphorylation sites. For example, there are 649979 ST-sites in 10195 proteins with predicted signal peptides (with any of the taxonomic versions of SIGNALP 3.0 [42]). pkaPS generates hits for 56970 sites (8.8%), a considerably lower value than that for the full proteome.

**Figure 7:**
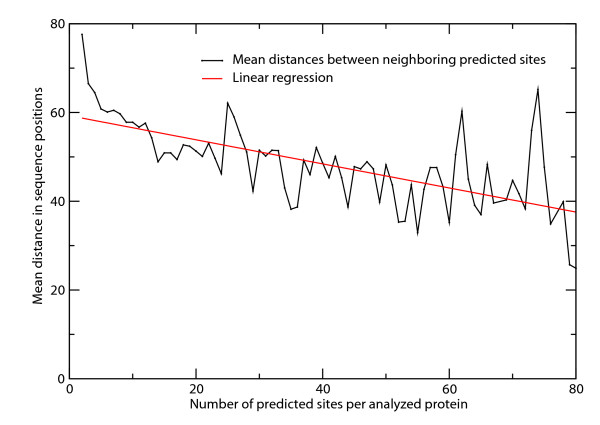
**Mean distances between pairs of neighboring predicted sites depending on the total number of predicted sites in the query proteins**. The red line displays the linear regression (y = 59.3 - 0.271x; R = -0.66) calculated using these data points.

Proteins with many serines/threonines are likely to have multiple PKA-dependent phosphorylation site predictions. For the human proteome, we find that the more sites are predicted per proteins, the smaller the mean distance between them (Figure 7). This confirms the trends observed in Figure 2 that proteins with many phosphorylation sites tend to pack these closely together into unified serine/threonine-rich regions.

The probability of wrongly predicting a site within a generally non-phosphorylated protein appears to dramatically increase with the number of S/T sites in its sequence. Among the 40877 sequences that are included in the retrieved file, only 4860 entries (11.9%) do not have a single predicted site for PKA-dependent phosphorylation. This result strongly emphasizes the main difficulty of predicting post-translational modifications that can occur in a query protein with multiple suitable serines/threonines. In such cases, a single false-positive site may be responsible for an incorrect assignment of the entire protein.

An increase in prediction accuracy may be obtained by grouping predicted entries together to clusters of related sequences. Naturally, predictions for a single post-translational modification can be considered more reliable if they are frequently observed in a protein family as opposed to a lone protein sequence [43] although this trend is not absolute even for closely related homologues [17,44]. To test the pkaPS tool on families of homologous sequences, the human proteome was clustered into 14674 groups with the MCL algorithm [45] (as implemented within the ANNOTATOR suite [46]) and each group was analyzed with the predictor separately. Many of these clusters only contained a single or a few sequences. All groups with less than 20 entries were removed from further considerations. The remaining 182 clusters were sorted according to the ratio between predicted serines/threonines and the total number of these residues in the cluster. The 20 clusters with the highest ratio are listed in Table 4. The good performance of the predictor is supported by the fact that families of proteins known to be good PKA targets occupy the top ranks in this listing. In addition to known phosphorylated sequence classes (e.g. histone H2A), there are also entirely uncharacterized groups of proteins that deserve experimental analysis. It is remarkable that this list does not contain any obviously false-positive sequence family.

**Table 4:** Prediction of the clustered human proteome.

Cluster number	Total entries	Predicted S/T (%)	Predicted S/T	Predicted entries (%)	Predicted entries	Common domains/superfamily
171	21	39.7	52	100	21	High mobility group
88	32	36.2	141	100	32	Histone H2A
43	48	30.8	518	91.7	44	Splicing factor, predicted RNA-binding
84	34	29	249	100	34	TAFII28-like
109	27	27.3	81	100	27	Unknown
72	36	26.4	101	94.4	34	GAGE protein
131	24	24.7	80	87.5	21	Ribosomal protein L21e
123	25	22.8	146	100	25	Ribosomal protein L22
172	21	22.5	108	100	21	Histone H2B
33	59	21.6	390	98.3	58	Cyclophilin-like
94	30	21.1	111	93.3	28	Histones H3/H4
105	27	20.9	88	96.3	26	KRAB domain
7	136	20	1300	96.3	131	GTPase-activating protein
10	123	19.7	412	84.6	104	Unknown
115	26	19.6	126	100	26	60S ribosomal protein L6
154	22	18.7	94	100	22	HIV-1 Vpr-binding, High mobility group
79	35	17.5	206	91.4	32	Ras GTPase-activating protein
38	52	17.4	287	92.3	48	Septins
153	22	16.3	105	77.3	17	RNA-binding protein TIA-1/TIAR (RRM superfamily)
160	22	16.1	465	100	22	Uncharacterized conserved protein (KOG4791)

The table shows the 20 clusters with the best ratio between predicted and total S/T-sites. The column to the right displays a summary of the cluster with respect to the most common domains found using the CDD [85] and PFAM [86] databases (e-value cutoff of 0.01). Lower cluster numbers indicate clusters with more sequences included.

The prediction of phosphorylation sites in ribosomal proteins such as L21e, L22 and L6 deserves special attention in context with the recent discovery of phosphorylation of some ribosomal proteins by specific kinases (such as the ribosomal protein S6 kinase (S6K)) and the important biological role of this phosphorylation [47,48].

#### Description of the associated WWW site

Supplementary data as well as the pkaPS WWW-server are available at the Mendel WWW-site [49]. The pkaPS server currently accepts up to 500 sequences in fasta-format (with no more 10000 S/T residues). For analyzing larger sets, we recommend contacting the authors. In interpreting the results, we advise to consider scores above 0 as good predictions; the twilight zone limit is -0.5. The predictor pkaPS analyzes the capability of the query sequence to productively interact with PKA. Additional information should be gathered from the literature or from predictors for other sequence properties to decide whether the prediction is not a hidden signal and makes sense in the biological context of the query. Additionally, we provide (i) access to the learning set, (ii) detailed results of the self-consistency and the neighbor-jackknife tests, (iii) downloads of the predictions for the human proteome both in plain and MCL-clustered forms [45].

## Discussion

Despite considerable algorithmic advances in the field, none of the prediction tools for PKA-dependent phosphorylation previously described in the literature achieves specificity and sensitivity rates both above 90%. In our view, several biological and computational aspects contribute to this development. Among them, there are several problems: (a) with the availability of experimental data, (b) with serine/threonine-rich regions, (c) with the incorporation of the available physico-chemical and biological knowledge into the scoring function used to discriminate between productively interacting substrates from non-permissive sequence stretches, (d) with the issue of accessibility of the phosphorylation motif within the protein's three-dimensional structure (intrinsically disordered regions surrounding the site) [27,50] and (e) with the issue of hidden signals (proteins that would be phosphorylated if they were in contact with PKA but which never have the appropriate biological context during their life cycle) [41].

With regard to point (a), little experimental data is available for most kinases with regard to the sequence variability of substrates, structural detail of kinase-substrate complexes, kinetic or energetic aspects of the interaction. Only a few kinases including PKA are reasonably well studied in this respect. For example, the number of sequentially dissimilar substrate sequences for the reliably parameterization of the profile term was estimated at least 200 in [50]. This number is reached for PKA even in the neighbor jackknife test (among the 239 sequence examples, the number of excluded sequences has never been above 10) and the results of this test show that stable profile parameterization has almost been achieved.

The issue of many serines/threonines in the sequence (b) is especially challenging since ST-rich regions are common in intra- and extracellular proteins. To detect phosphorylated proteins on a large-scale basis, every single potential site in a sequence must be taken into consideration. If ***S***_***p ***_(measured as value between 0 and 1) is the rate of correct rejection of a site and if there are *n *serine/threonine residues in a query sequence, the specificity of the task for classification of query proteins decreases significantly (to Spn
 MathType@MTEF@5@5@+=feaafiart1ev1aaatCvAUfKttLearuWrP9MDH5MBPbIqV92AaeXatLxBI9gBaebbnrfifHhDYfgasaacH8akY=wiFfYdH8Gipec8Eeeu0xXdbba9frFj0=OqFfea0dXdd9vqai=hGuQ8kuc9pgc9s8qqaq=dirpe0xb9q8qiLsFr0=vr0=vr0dc8meaabaqaciaacaGaaeqabaqabeGadaaakeaaieWacqWFtbWudaqhaaWcbaGae8hCaahabaGae8NBa4gaaaaa@30D6@ << 1). Considering the difficulties associated with prediction of potentially multiple sites in ST-rich regions, it is clear that very high accuracies are needed if such algorithms are to be applied routinely on a large-scale proteome basis.

The incorporation of the heterogeneous knowledge about the PKA-substrate protein relationship into the scoring function (issue c) is a non-trivial problem. Experimental reports usually do not provide the knowledge in the form that is necessary for formulating algorithms. There are two ways to deal with this problem – either to take the information as is and to hope that machine learning procedures filter the aspects of the data that are relevant for prediction, or to formulate a physically reasonable model of productive binding events with the kinase directly. Machine learning approaches have shown their usability in a variety of applications, especially in cases where lots of uniform data are available. The classical example is SIGNALP, for the derivation of which learning sets in the size of thousands of substrate proteins were collected [42].

In many other biological applications, the data situation is by far not that comfortable. In such circumstances, the usage of machine learning software packages as "black boxes" for autonomous extraction of score function parameters without human interference and without explicitly considering the physico-chemical and biological realities of the problem under study can become dangerous. In their letter to the editors of the Biophysics Journal in 1994 [51], Frank Darius and Raul Rojas analyze the difficulties arising from the discrepancy between the very high dimension of the parameter space in modern machine learning approaches and scarce data when exemplarily criticizing an alternative signalpeptide predictor. To summarize, the problem is that the calculated parameters are not reliable and it is not clear whether the correlations found are numerical noise of the data or biologically meaningful. If the data are scarce and no one tells the "black box" how the substrate protein interacts with the receptor, then the box would indeed need to be a "magic box" to know about it in order to pick the correct significant parameters.

As a direct consequence, human involvement and additional biological knowledge are indispensable for dimensionality reduction. In contrast to machine learning approaches, a physically justified model of productive binding with the kinase already provides a reasonable analytical form of scoring function terms. In this context, it is not so important whether this form can be further improved. We wish to emphasize that the number of parameters to be determined with the help of learning data is dramatically reduced.

In our approach, we consequently follow these considerations and try to incorporate all biologically relevant information into the analytical form of the prediction function. For example, it is utterly important that among all sequence positions, which carry relevant information, as many as possible are considered for the prediction procedure. In the case of PKA, we found the region to occupy the segment -18...+23. Also, we have very few parameters: a profile term over 13 positions centered around the putative site and 14 physical property terms *T*_*j *_(typically involving 3 parameters: the mean and the standard deviation of an amino acid index averaged over some sequence region as well as a weight factor for the whole term). Especially the latter set of parameters is determined with high significance given the 239 positive examples. We think that the simplicity of our algorithm is its big strength since the output of the decision function clearly indicates what kind of property supports or prevents the prediction of a query as PKA substrate. In the process of predictor development, human interference can assure that only the biologically meaningful among the significant correlations enter the decision function.

The influence of the structure of the protein on the accessibility of the phosphorylation motif to the kinase (issue d) is difficult to estimate at present. By demanding an excess of hydrophilic, small and flexible residues in the region -18...+23 with the physical property terms, it becomes quite unlikely that a sequence region hit by our predictor is actually part of a 3D structure but rather represents an intrinsically disordered segment. Nevertheless, it cannot be excluded that sequence stretches that are not included in the motif definition might cause the entire protein to fold in such a way that the potential phosphorylation site is not accessible to its modifying kinase.

Finally, the cellular context is important (issue e). In the case of some translocation signals, it has been experimentally shown that even the presence of an *in vivo *functional motif does not mean that the carrying protein is also imported into the corresponding subcellular compartment [41]. This discovery of hidden sequence signals highlights the significance of cellular hierarchies for small functional protein motifs. Hence, current phosphorylation predictors including pkaPS do not really predict phosphorylation, but the potential of a sequence stretch to interact productively with the modifying kinase. This seemingly small detail may appear negligible but it is important to be considered in all predictions. E.g. a targeting signal located far away from a functional phosphorylation site on the same protein may lead to a removal from the cellular compartment of the respective kinase, thereby overriding the phosphorylation motif. This means that the analyzed motif is not the sole sequence stretch on the protein which is responsible for the modification.

These considerations mean that a phosphorylation predictor is not the only source of information that must be consulted when evaluating the phosphorylation state of a protein. Moreover, the number of apparently wrong predictions (if only the physiologically relevant cases are counted) of an algorithm is not only determined by the imperfection of its design since the predictor focuses on the query sequence stretch. Hence, even if all permissive amino acid permutations of the substrate motif are known, the theoretical accuracy of any post-translational modification predictor will have an upper limit clearly below 100%.

## Conclusion

The refinement of the PKA phosphporylation motif showed that approximately 20 sequence positions flanking the phosphorylated residue on both sides are restricted in their sequence variability. The conserved physical pattern can be rationalized in terms of a qualitative binding model with the catalytic cleft of the protein kinase A. The pkaPS predictor based on this motif description confidently discriminates PKA phosphorylation sites from serines/threonines with non-permissive sequence environments (sensitivity of ~96% at a specificity of ~94%).

## Methods

### Learning set construction

UNIPROT [52-54] accessions and positions of sites which are phosphorylated via PKA were retrieved from the Phospho.ELM database version 4.0 [55]. Subsequently, an alignment was generated which contains the 81-residue long sequences that span the phosphorylated residue in addition to the 40 flanking amino acids on each side. Positions outside of the N- or C-terminal ends were treated as non-occupied (without amino acid) in further calculations. The original sequences of the substrate proteins were obtained from the UNIPROT database [56]. Initiator methionines were removed according to the 1.29Å rule [57,58].

Previous analyses of typical annotation errors in databases [17,20,22,42] emphasized the importance of learning set curation. As expected, a couple of entries were inaccurate or had unclear verifications. Therefore, the following modifications were introduced (protein sequences are indicated by UNIPROT accession numbers):

• The first phosphorylation site of P02646 is actually a double site that lies at positions 22/23 instead of position 20 [59,60].

• Position 137 of the Casein-B precursor sequence (P02666) contains arginine, not serine. According to the paper where the experimental verification is reported [61], phosphorylation occurs in a "variant B" which contains this mutation.

• The experimental verification in the paper cited for the entry P11168 was actually performed for the rat (RINm5F cell line), not the human protein [62]. As a consequence, the entry was replaced by the corresponding rat sequence P12336, with the reported phosphorylation sites located at positions 489, 501, 503 and 510.

• The exact localization of the PKA-dependent phosphorylation sites in the PTH/PTHrP type I receptor (P25107) was performed using the rat protein, not the one from opossum [63]. Therefore, P25107 was replaced by P25961 (positions 491, 473 and 475).

• Phosphorylation of the sites in P00698 appears to occur only in the denaturized protein [64]. As the experimental verification status of the entry is not entirely clear, it was excluded from the dataset.

• Entry P13280 is removed from the dataset because the experimental verification for this extremely unusual motif is unclear [65].

• Serine 259 from P04049 is phosphorylated [66] but not listed in Phosph.ELM and was, therefore, added subsequently.

• According to the corresponding paper [67] and the UNIPROT entry, the phosphorylated residue of P20020 lies at position 1216 and not 1178.

• Phosphorylation of γ-aminobutyric-acid receptor β1 is originally reported for the mouse protein instead of the human counterpart [68]. As a consequence, entry P18505 was replaced by P50571.

• The reported phosphorylation sites for P32245 are only proposed to be potential sites for PKA and GRK. They actually lack any direct experimental verification [69] for PKA-dependent phosphorylation. The corresponding entries were removed from the learning set as a consequence.

• Phosphorylation of the metallopeptidase EP24.15 is reported for the rat instead of the human protein [70]. Entry P52888 was, therefore, replaced by P24155 including the correct location of the phosphorylated serine.

• The phosphorylated serine in P07101 is located at position 71, not 40. Although the original paper reports Ser40 as site [71], the PKA-motif is shifted in direction of the C-terminus in the UNIPROT entry as a result of additional N-terminal regions from splice variants. Moreover, the experimental verification was performed using the rat protein, and the phosphorylated serine is annotated as "by similarity" in the UNIPROT sequence. Hence, the entry was removed from the learning set.

• P01233 can theoretically be phosphorylated at three sites. However, the post-translational modification states depend on whether the implicated β-subunit is free and in its native form [72]. Therefore, the corresponding entry was removed from the learning set.

To generate a set of negative examples, the references of a set of learning set sequences were screened. If it could be deduced that the phosphorylated S/T-sites reported in a publication were the sole amino acids that are modified by PKA, then all remaining S/T-sites were added to the set of negative examples.

The final learning sets consist of 143 sequences with 239 phosphorylated sites and 28 sequences with 1026 non-phosphorylated serines and threonines. Although the set of positive examples contains entries from various taxonomical groups, it is mostly centered on mammalian species. Around one half of the 239 sites originate from H. sapiens (120 sites). Together with the other mammalian entries (93 sites), they make up 89% of the learning set. From the remaining entries, 21 originate from other metazoan species, and only 5 are from yeast and viridiplantae.

It should be noted that phosphorylation frequently occurs at multiple sites of the same substrate protein (in contrast to several other posttranslational modifications) and this is reflected in the learning set. From 143 sequences included in the set of positive examples, more than one third has more than one verified serine or threonine residue. As a consequence, two thirds of the phosphorylated sites in the learning set originate from proteins with multiple modifications. The corresponding distribution of the numbers of phosphorylation sites per protein (shown in Table 5) seems to fall exponentially.

**Table 5:** Distribution of the number of phosphorylated sites per sequence in the learning set.

Sites per sequence	N_sequences_	%	% (cum.)	N_sites_	%	% (cum.)
1	88	61.5	61.5	88	36.8	36.8
2	30	21.0	82.5	60	25.1	61.9
3	15	10.5	93.0	45	18.8	80.7
4	7	4.9	97.9	28	11.7	92.4
5	1	0.7	98.6	5	2.1	94.5
6	1	0.7	99.3	6	2.5	97.0
7	1	0.7	100.0	7	3.0	100.0

Total	143	100.0	100.0	239	100.0	100.0

Positive examples in the dataset contain up to seven sites per sequence. Although approximately two thirds (88) of all entries (143) contain only one phosphorylated serine or threonine, this accounts for just slightly more than one third of the total number of included sites (239). It should be noted that, for many proteins, additional phosphorylation sites might have been undetected so far.

### Sequence analysis part 1. Redundancy removal

The phosphorylated serines/threonines of a learning set together with their flanking sequences are represented in a gapless multiple alignment of n_pos _= 81 positions. The n_seq _= 239 phosphorylated sites occupy a single column that acts as reference location with an assigned position number of 0. Sequence positions further N-terminally have negative values, positions further C-terminally have positive values.

To remove redundancy from over-represented sequence sets in the learning alignment [73], we used a technique similar to the "sum of mismatches"-method from Vingron and Argos [74,75]. The central consideration is that the higher the similarity of a sequence is to all remaining sequences in the alignment, the lower its weight ***w ***should be. Here, the number of identical residues between two sequences ***k ***and ***i ***is chosen as a distance measure. For each sequence ***k***, the weight ***w***_***k ***_is calculated using Kronecker's delta according to equation 1. The value *γ *is obtained from the normalization to ∑***w_k _***= ***n_seq_***. The use of a modified version of the original Vingron and Argos method is due to the disproportionally high weights that the original method assigns to sequences with many non-amino acid positions such as ones which are outside of the sequence for sites close to either the N- or C-termini.

wk=γnpos∑l=1npos∑i=1nseqδ(a(k,l),a(i,l))     (1)
 MathType@MTEF@5@5@+=feaafiart1ev1aaatCvAUfKttLearuWrP9MDH5MBPbIqV92AaeXatLxBI9gBaebbnrfifHhDYfgasaacH8akY=wiFfYdH8Gipec8Eeeu0xXdbba9frFj0=OqFfea0dXdd9vqai=hGuQ8kuc9pgc9s8qqaq=dirpe0xb9q8qiLsFr0=vr0=vr0dc8meaabaqaciaacaGaaeqabaqabeGadaaakeaaieWacqWF3bWDdaWgaaWcbaGae83AaSgabeaakiabg2da9GGaciab+n7aNnaalaaabaGae8NBa42aaSbaaSqaaiab=bhaWjab=9gaVjab=nhaZbqabaaakeaadaaeWbqaamaaqahabaGae4hTdqMaeiikaGIae8xyaeMaeiikaGIae83AaSMaeiilaWIae8hBaWMaeiykaKIaeiilaWIae8xyaeMaeiikaGIae8xAaKMaeiilaWIae8hBaWMaeiykaKIaeiykaKcaleaacqWFPbqAcqGH9aqpcqaIXaqmaeaacqWFUbGBdaWgaaadbaGae83CamNae8xzauMae8xCaehabeaaa0GaeyyeIuoaaSqaaiab=XgaSjabg2da9iabigdaXaqaaiab=5gaUnaaBaaameaacqWFWbaCcqWFVbWBcqWFZbWCaeqaaaqdcqGHris5aaaakiaaxMaacaWLjaWaaeWaaeaacqaIXaqmaiaawIcacaGLPaaaaaa@63F3@

### Sequence analysis part 2. Derivation of physical property characteristics

To assess physical and chemical requirements at specific motif positions, we make use of 20-dimensional property vectors ***v ***which assign characteristic values *v*_*a *_to each amino acid *a*. These values have been measured in various experimental setups and quantify amino acid properties such as e.g. hydrophobicity, volume or charge. We used a pre-compiled property database [23,28] for the motif analysis. Here, single property vectors are typically specified by short identifiers such as EISD840101. We use only properties which have defined values for all 20 amino acids.

One means of detecting amino acid requirements for a sequence position ***l ***is to compare property mean values v¯
 MathType@MTEF@5@5@+=feaafiart1ev1aaatCvAUfKttLearuWrP9MDH5MBPbIqV92AaeXatLxBI9gBaebbnrfifHhDYfgasaacH8akY=wiFfYdH8Gipec8Eeeu0xXdbba9frFj0=OqFfea0dXdd9vqai=hGuQ8kuc9pgc9s8qqaq=dirpe0xb9q8qiLsFr0=vr0=vr0dc8meaabaqaciaacaGaaeqabaqabeGadaaakeaaieWacuWF2bGDgaqeaaaa@2E41@(*l*) with expected mean values v¯
 MathType@MTEF@5@5@+=feaafiart1ev1aaatCvAUfKttLearuWrP9MDH5MBPbIqV92AaeXatLxBI9gBaebbnrfifHhDYfgasaacH8akY=wiFfYdH8Gipec8Eeeu0xXdbba9frFj0=OqFfea0dXdd9vqai=hGuQ8kuc9pgc9s8qqaq=dirpe0xb9q8qiLsFr0=vr0=vr0dc8meaabaqaciaacaGaaeqabaqabeGadaaakeaaieWacuWF2bGDgaqeaaaa@2E41@_*DB *_from biological databases. Significances can be assessed using Student's *t*-distribution with the help of the property value dispersion σ(*l*). The values ***p***_***a ***_represent the database occurrences of amino acid type ***a ***calculated on the basis of UNIREF.

v¯(l)=∑kwkva(k,l)∑kwk     (2)
 MathType@MTEF@5@5@+=feaafiart1ev1aaatCvAUfKttLearuWrP9MDH5MBPbIqV92AaeXatLxBI9gBaebbnrfifHhDYfgasaacH8akY=wiFfYdH8Gipec8Eeeu0xXdbba9frFj0=OqFfea0dXdd9vqai=hGuQ8kuc9pgc9s8qqaq=dirpe0xb9q8qiLsFr0=vr0=vr0dc8meaabaqaciaacaGaaeqabaqabeGadaaakeaaieWacuWF2bGDgaqeaiabcIcaOiab=XgaSjabcMcaPiabg2da9maalaaabaWaaabuaeaacqWF3bWDdaWgaaWcbaGae83AaSgabeaakiab=zha2naaBaaaleaacqWFHbqycqGGOaakcqWFRbWAcqGGSaalcqWFSbaBcqGGPaqkaeqaaaqaaiab=TgaRbqab0GaeyyeIuoaaOqaamaaqafabaGae83DaC3aaSbaaSqaaiab=TgaRbqabaaabaGae83AaSgabeqdcqGHris5aaaakiaaxMaacaWLjaWaaeWaaeaacqaIYaGmaiaawIcacaGLPaaaaaa@4B4B@

v¯DB=∑avapa∑apa     (3)
 MathType@MTEF@5@5@+=feaafiart1ev1aaatCvAUfKttLearuWrP9MDH5MBPbIqV92AaeXatLxBI9gBaebbnrfifHhDYfgasaacH8akY=wiFfYdH8Gipec8Eeeu0xXdbba9frFj0=OqFfea0dXdd9vqai=hGuQ8kuc9pgc9s8qqaq=dirpe0xb9q8qiLsFr0=vr0=vr0dc8meaabaqaciaacaGaaeqabaqabeGadaaakeaaieWacuWF2bGDgaqeamaaBaaaleaacqWFebarcqWFcbGqaeqaaOGaeyypa0ZaaSaaaeaadaaeqbqaaiab=zha2naaBaaaleaacqWFHbqyaeqaaOGae8hCaa3aaSbaaSqaaiab=fgaHbqabaaabaGae8xyaegabeqdcqGHris5aaGcbaWaaabuaeaacqWFWbaCdaWgaaWcbaGae8xyaegabeaaaeaacqWFHbqyaeqaniabggHiLdaaaOGaaCzcaiaaxMaadaqadaqaaiabiodaZaGaayjkaiaawMcaaaaa@44D4@

σ(l)=∑kwk(va(k,l)−v¯(l))2∑kwk−1     (4)
 MathType@MTEF@5@5@+=feaafiart1ev1aaatCvAUfKttLearuWrP9MDH5MBPbIqV92AaeXatLxBI9gBaebbnrfifHhDYfgasaacH8akY=wiFfYdH8Gipec8Eeeu0xXdbba9frFj0=OqFfea0dXdd9vqai=hGuQ8kuc9pgc9s8qqaq=dirpe0xb9q8qiLsFr0=vr0=vr0dc8meaabaqaciaacaGaaeqabaqabeGadaaakeaaiiGacqWFdpWCcqGGOaakieWacqGFSbaBcqGGPaqkcqGH9aqpdaGcaaqaamaalaaabaWaaabuaeaacqGF3bWDdaWgaaWcbaGae43AaSgabeaakmaabmaabaGae4NDay3aaSbaaSqaaiab+fgaHjabcIcaOiab+TgaRjabcYcaSiab+XgaSjabcMcaPaqabaGccqGHsislcuGF2bGDgaqeaiabcIcaOiab+XgaSjabcMcaPaGaayjkaiaawMcaamaaCaaaleqabaGaeGOmaidaaaqaaiab+TgaRbqab0GaeyyeIuoaaOqaamaaqafabaGae43DaC3aaSbaaSqaaiab+TgaRbqabaGccqGHsislcqaIXaqmaSqaaiab+TgaRbqab0GaeyyeIuoaaaaaleqaaOGaaCzcaiaaxMaadaqadaqaaiabisda0aGaayjkaiaawMcaaaaa@55C7@

A more sensitive method involves the calculation of the correlation coefficient *R(l) *between the property values *v*_*a *_and the observed amino acid counts *c*(*a, l*) at an alignment position *l*. The underlying consideration is that amino acids with high values *v*_*a *_of a required property should occur more frequently than residues with hindering, low property values (or vice versa).

R(l)=20∑avac(a,l)−∑ava∑ac(a,l)(20∑ava2−(∑ava)2)(20∑ac(a,l)2−(∑ac(a,l))2)     (5)
 MathType@MTEF@5@5@+=feaafiart1ev1aaatCvAUfKttLearuWrP9MDH5MBPbIqV92AaeXatLxBI9gBaebbnrfifHhDYfgasaacH8akY=wiFfYdH8Gipec8Eeeu0xXdbba9frFj0=OqFfea0dXdd9vqai=hGuQ8kuc9pgc9s8qqaq=dirpe0xb9q8qiLsFr0=vr0=vr0dc8meaabaqaciaacaGaaeqabaqabeGadaaakeaaieWacqWFsbGucqGGOaakcqWFSbaBcqGGPaqkcqGH9aqpdaWcaaqaaiabikdaYiabicdaWmaaqafabaGae8NDay3aaSbaaSqaaiab=fgaHbqabaGccqWFJbWycqGGOaakcqWFHbqycqGGSaalcqWFSbaBcqGGPaqkaSqaaiab=fgaHbqab0GaeyyeIuoakiabgkHiTmaaqafabaGae8NDay3aaSbaaSqaaiab=fgaHbqabaaabaGae8xyaegabeqdcqGHris5aOWaaabuaeaacqWFJbWycqGGOaakcqWFHbqycqGGSaalcqWFSbaBcqGGPaqkaSqaaiab=fgaHbqab0GaeyyeIuoaaOqaamaakaaabaWaaeWaaeaacqaIYaGmcqaIWaamdaaeqbqaaiab=zha2naaDaaaleaacqWFHbqyaeaacqaIYaGmaaaabaGae8xyaegabeqdcqGHris5aOGaeyOeI0YaaeWaaeaadaaeqbqaaiab=zha2naaBaaaleaacqWFHbqyaeqaaaqaaiab=fgaHbqab0GaeyyeIuoaaOGaayjkaiaawMcaamaaCaaaleqabaGaeGOmaidaaaGccaGLOaGaayzkaaWaaeWaaeaacqaIYaGmcqaIWaamdaaeqbqaaiab=ngaJjabcIcaOiab=fgaHjabcYcaSiab=XgaSjabcMcaPmaaCaaaleqabaGaeGOmaidaaaqaaiab=fgaHbqab0GaeyyeIuoakiabgkHiTmaabmaabaWaaabuaeaacqWFJbWycqGGOaakcqWFHbqycqGGSaalcqWFSbaBcqGGPaqkaSqaaiab=fgaHbqab0GaeyyeIuoaaOGaayjkaiaawMcaamaaCaaaleqabaGaeGOmaidaaaGccaGLOaGaayzkaaaaleqaaaaakiaaxMaacaWLjaWaaeWaaeaacqaI1aqnaiaawIcacaGLPaaaaaa@867D@

The statistical significance of *R *(*l*) can be calculated using the decision criterion [76]:

tα=Rnv−31−R2     (6)
 MathType@MTEF@5@5@+=feaafiart1ev1aaatCvAUfKttLearuWrP9MDH5MBPbIqV92AaeXatLxBI9gBaebbnrfifHhDYfgasaacH8akY=wiFfYdH8Gipec8Eeeu0xXdbba9frFj0=OqFfea0dXdd9vqai=hGuQ8kuc9pgc9s8qqaq=dirpe0xb9q8qiLsFr0=vr0=vr0dc8meaabaqaciaacaGaaeqabaqabeGadaaakeaaieWacqWF0baDdaWgaaWcbaacciGae4xSdegabeaakiabg2da9maalaaabaGae8Nuai1aaOaaaeaacqWFUbGBdaWgaaWcbaGae8NDayhabeaakiabgkHiTiabiodaZaWcbeaaaOqaamaakaaabaGaeGymaeJaeyOeI0Iae8Nuai1aaWbaaSqabeaacqaIYaGmaaaabeaaaaGccaWLjaGaaCzcamaabmaabaGaeGOnaydacaGLOaGaayzkaaaaaa@3F53@

*t*_*α *_is the argument of the Student's distribution function for a one-sided criterion with the confidence level α, and 3 stands for the number of conditions (two for the linear regression and one for the sum of all residue type frequencies being unity).

We employ Fisher's test for the detection of inter-positional correlations. Here, the sum of the squared variances *s*(*l*_*i*_) for all *n*_*pos *_isolated positions *l*_*i *_is compared to the squared variance *σ*(*l*_*1*_*,l*_*2*_*,...,l*_*npos*_) of the combined positions [20-24,26]:

F(l1,l2,...,lnpos)=∑i=1nopsσ(li)2σ(l1,l2,...,lnpos)2     (7)
 MathType@MTEF@5@5@+=feaafiart1ev1aaatCvAUfKttLearuWrP9MDH5MBPbIqV92AaeXatLxBI9gBaebbnrfifHhDYfgasaacH8akY=wiFfYdH8Gipec8Eeeu0xXdbba9frFj0=OqFfea0dXdd9vqai=hGuQ8kuc9pgc9s8qqaq=dirpe0xb9q8qiLsFr0=vr0=vr0dc8meaabaqaciaacaGaaeqabaqabeGadaaakeaaieWacqWFgbGrdaqadaqaaiab=XgaSnaaBaaaleaacqaIXaqmaeqaaOGaeiilaWIae8hBaW2aaSbaaSqaaiabikdaYaqabaGccqGGSaalcqGGUaGlcqGGUaGlcqGGUaGlcqGGSaalcqWFSbaBdaWgaaWcbaGae8NBa42aaSbaaWqaaiab=bhaWjab=9gaVjab=nhaZbqabaaaleqaaaGccaGLOaGaayzkaaGaeyypa0ZaaSaaaeaadaaeWbqaaGGaciab+n8aZnaabmaabaGae8hBaW2aaSbaaSqaaiab=LgaPbqabaaakiaawIcacaGLPaaadaahaaWcbeqaaiabikdaYaaaaeaacqWFPbqAcqGH9aqpcqaIXaqmaeaacqWFUbGBdaWgaaadbaGae83Ba8Mae8hCaaNae83Camhabeaaa0GaeyyeIuoaaOqaaiab+n8aZnaabmaabaGae8hBaW2aaSbaaSqaaiabigdaXaqabaGccqGGSaalcqWFSbaBdaWgaaWcbaGaeGOmaidabeaakiabcYcaSiabc6caUiabc6caUiabc6caUiabcYcaSiab=XgaSnaaBaaaleaacqWFUbGBdaWgaaadbaGae8hCaaNae83Ba8Mae83CamhabeaaaSqabaaakiaawIcacaGLPaaadaahaaWcbeqaaiabikdaYaaaaaGccaWLjaGaaCzcamaabmaabaGaeG4naCdacaGLOaGaayzkaaaaaa@6EB0@

The obtained F-value follows an F-distribution with *n*_*seq *_– 1 degrees of freedom [76]. For weighted sequences, *n*_*seq *_needs to be replaced by the sum of the weights of all sequences that are included in *F*-value calculation.

Mean values and standard deviations (equations 2, 3 and 4) as well as property correlations (equations 5 and 6) and F-tests (equation 7) have been routinely used in the derivation of the physical property pattern surrounding the phosphorylation sites (see first three sections of Results). In the Results, we often write v¯
 MathType@MTEF@5@5@+=feaafiart1ev1aaatCvAUfKttLearuWrP9MDH5MBPbIqV92AaeXatLxBI9gBaebbnrfifHhDYfgasaacH8akY=wiFfYdH8Gipec8Eeeu0xXdbba9frFj0=OqFfea0dXdd9vqai=hGuQ8kuc9pgc9s8qqaq=dirpe0xb9q8qiLsFr0=vr0=vr0dc8meaabaqaciaacaGaaeqabaqabeGadaaakeaaieWacuWF2bGDgaqeaaaa@2E41@, **σ**, ***R ***and ***F ***without positional arguments when we describe the positions in the text.

### Details of the prediction methodology

Each prediction produces a score S that is composed of a profile term *S*_*profile *_and a physico-chemical penalty value *S*_*ppt*_.

***S ***= ***S***_***profile ***_+ ***S***_***ppt ***_    (8)

The query sequence is predicted if the score is ≥ than a predefined threshold *b*. We chose a threshold of ***b ***= 0 for the prediction of PKA-dependent phosphorylation and ***b ***= -0.5 for the twilight zone (see Results). The profile term is calculated using the PSIC algorithm [77], a method that provides sequence- and alignment position-specific weights, to remove redundancy from homologous sequences that originate from over-represented protein families. Note that the redundancy removal for the physical property calculation was carried out differently with a modification of the Vingron and Argos procedure [74,75] (see Sequence analysis part 1 in Methods). As different motif positions generally have different importance for substrate binding efficiency, the profile value contributions *S*_*j*_(*a*(*l*_*j*_)) of amino acids *a *at positions *l*_*j *_are weighted using factors *α*_***profile, j ***_For a profile that consists of *n*_*pos *_positions, the term *S*_*profile *_can be expressed as:

Sprofile=∑j=1nposαprofile,jSj(a(lj))     (9)
 MathType@MTEF@5@5@+=feaafiart1ev1aaatCvAUfKttLearuWrP9MDH5MBPbIqV92AaeXatLxBI9gBaebbnrfifHhDYfgasaacH8akY=wiFfYdH8Gipec8Eeeu0xXdbba9frFj0=OqFfea0dXdd9vqai=hGuQ8kuc9pgc9s8qqaq=dirpe0xb9q8qiLsFr0=vr0=vr0dc8meaabaqaciaacaGaaeqabaqabeGadaaakeaaieWacqWFtbWudaWgaaWcbaGae8hCaaNae8NCaiNae83Ba8Mae8NzayMae8xAaKMae8hBaWMae8xzaugabeaakiabg2da9maaqahabaacciGae4xSde2aaSbaaSqaaiab=bhaWjab=jhaYjab=9gaVjab=zgaMjab=LgaPjab=XgaSjab=vgaLjabcYcaSiab=PgaQbqabaaabaGae8NAaOMaeyypa0JaeGymaedabaGae8NBa42aaSbaaWqaaiab=bhaWjab=9gaVjab=nhaZbqabaaaniabggHiLdGccqWFtbWudaWgaaWcbaGae8NAaOgabeaakmaabmaabaGae8xyae2aaeWaaeaacqWFSbaBdaWgaaWcbaGae8NAaOgabeaaaOGaayjkaiaawMcaaaGaayjkaiaawMcaaiaaxMaacaWLjaWaaeWaaeaacqaI5aqoaiaawIcacaGLPaaaaaa@5F50@

The total penalty *S*_*ppt *_is simply the sum of all *n*_*penalties *_penalty terms *T*_*j*_, where each *T*_*j *_reflects a piece of the acquired knowledge about substrate binding requirements in the motif region. The height of the penalty can be adjusted using the corresponding weight factor *α*_***ppt, j***_.

Sppt=∑j=1npenaltiesαppt,jTj     (10)
 MathType@MTEF@5@5@+=feaafiart1ev1aaatCvAUfKttLearuWrP9MDH5MBPbIqV92AaeXatLxBI9gBaebbnrfifHhDYfgasaacH8akY=wiFfYdH8Gipec8Eeeu0xXdbba9frFj0=OqFfea0dXdd9vqai=hGuQ8kuc9pgc9s8qqaq=dirpe0xb9q8qiLsFr0=vr0=vr0dc8meaabaqaciaacaGaaeqabaqabeGadaaakeaaieWacqWFtbWudaWgaaWcbaGae8hCaaNae8hCaaNae8hDaqhabeaakiabg2da9maaqahabaacciGae4xSde2aaSbaaSqaaiab=bhaWjab=bhaWjab=rha0jabcYcaSiab=PgaQbqabaaabaGae8NAaOMaeyypa0JaeGymaedabaGae8NBa42aaSbaaWqaaiab=bhaWjab=vgaLjab=5gaUjab=fgaHjab=XgaSjab=rha0jab=LgaPjab=vgaLjab=nhaZbqabaaaniabggHiLdGccqWFubavdaWgaaWcbaGae8NAaOgabeaakiaaxMaacaWLjaWaaeWaaeaacqaIXaqmcqaIWaamaiaawIcacaGLPaaaaaa@5653@

Each term *T*_*j *_has an associated property **v**_j _that represents the type of physico-chemical requirement, e.g. hydrophobicity or size. Its mean value in the query sequence v¯j(l1,...,lnpos)
 MathType@MTEF@5@5@+=feaafiart1ev1aaatCvAUfKttLearuWrP9MDH5MBPbIqV92AaeXatLxBI9gBaebbnrfifHhDYfgasaacH8akY=wiFfYdH8Gipec8Eeeu0xXdbba9frFj0=OqFfea0dXdd9vqai=hGuQ8kuc9pgc9s8qqaq=dirpe0xb9q8qiLsFr0=vr0=vr0dc8meaabaqaciaacaGaaeqabaqabeGadaaakeaaieWacuWF2bGDgaqeamaaBaaaleaacqWFQbGAaeqaaOGaeiikaGIae8hBaW2aaSbaaSqaaiabigdaXaqabaGccqGGSaalcqGGUaGlcqGGUaGlcqGGUaGlcqGGSaalcqWFSbaBdaWgaaWcbaGae8NBa42aaSbaaWqaaiab=bhaWjab=9gaVjab=nhaZbqabaaaleqaaOGaeiykaKcaaa@3FD0@ over a set of *n*_*pos *_motif positions *l*_*i*_, together with the respective mean value over the learning set v¯jLS(l1,...,lnpos)
 MathType@MTEF@5@5@+=feaafiart1ev1aaatCvAUfKttLearuWrP9MDH5MBPbIqV92AaeXatLxBI9gBaebbnrfifHhDYfgasaacH8akY=wiFfYdH8Gipec8Eeeu0xXdbba9frFj0=OqFfea0dXdd9vqai=hGuQ8kuc9pgc9s8qqaq=dirpe0xb9q8qiLsFr0=vr0=vr0dc8meaabaqaciaacaGaaeqabaqabeGadaaakeaaieWacuWF2bGDgaqeamaaBaaaleaacqWFQbGAdaWgaaadbaGae8htaWKae83uamfabeaaaSqabaGccqGGOaakcqWFSbaBdaWgaaWcbaGaeGymaedabeaakiabcYcaSiabc6caUiabc6caUiabc6caUiabcYcaSiab=XgaSnaaBaaaleaacqWFUbGBdaWgaaadbaGae8hCaaNae83Ba8Mae83CamhabeaaaSqabaGccqGGPaqkaaa@4250@, the learning set dispersion σjLS(l1,...,lnpos)
 MathType@MTEF@5@5@+=feaafiart1ev1aaatCvAUfKttLearuWrP9MDH5MBPbIqV92AaeXatLxBI9gBaebbnrfifHhDYfgasaacH8akY=wiFfYdH8Gipec8Eeeu0xXdbba9frFj0=OqFfea0dXdd9vqai=hGuQ8kuc9pgc9s8qqaq=dirpe0xb9q8qiLsFr0=vr0=vr0dc8meaabaqaciaacaGaaeqabaqabeGadaaakeaaiiGacqWFdpWCdaWgaaWcbaacbmGae4NAaO2aaSbaaWqaaiab+Xeamjab+nfatbqabaaaleqaaOGaeiikaGIae4hBaW2aaSbaaSqaaiabigdaXaqabaGccqGGSaalcqGGUaGlcqGGUaGlcqGGUaGlcqGGSaalcqGFSbaBdaWgaaWcbaGae4NBa42aaSbaaWqaaiab+bhaWjab+9gaVjab+nhaZbqabaaaleqaaOGaeiykaKcaaa@4288@ and a freely selectable parameter *t*_*j *_are used as a basis for calculation of *T*_*j*_.

We use two different types of penalties: (i) fixed ones and (ii) gauss-type penalties. Fixed penalties are simple penalties that are applied if the property mean value v¯j(l1,...,lnpos)
 MathType@MTEF@5@5@+=feaafiart1ev1aaatCvAUfKttLearuWrP9MDH5MBPbIqV92AaeXatLxBI9gBaebbnrfifHhDYfgasaacH8akY=wiFfYdH8Gipec8Eeeu0xXdbba9frFj0=OqFfea0dXdd9vqai=hGuQ8kuc9pgc9s8qqaq=dirpe0xb9q8qiLsFr0=vr0=vr0dc8meaabaqaciaacaGaaeqabaqabeGadaaakeaaieWacuWF2bGDgaqeamaaBaaaleaacqWFQbGAaeqaaOGaeiikaGIae8hBaW2aaSbaaSqaaiabigdaXaqabaGccqGGSaalcqGGUaGlcqGGUaGlcqGGUaGlcqGGSaalcqWFSbaBdaWgaaWcbaGae8NBa42aaSbaaWqaaiab=bhaWjab=9gaVjab=nhaZbqabaaaleqaaOGaeiykaKcaaa@3FD0@ in the query sequence exceeds the predefined threshold *t*_*j*_, without taking the learning set into account. *T*_*j *_is then either 0 or -1.

Whereas fixed *T*_*j *_only penalize the mere occurrence of potentially hindering amino acids, Gaussian-type penalties also take into account the level of deviation from property preferences at motif positions. To exclude sequences that strongly deviate from the derived consensus, the value of *T*_*j *_increases with the square of the difference between v¯j(l1,...,lnpos)
 MathType@MTEF@5@5@+=feaafiart1ev1aaatCvAUfKttLearuWrP9MDH5MBPbIqV92AaeXatLxBI9gBaebbnrfifHhDYfgasaacH8akY=wiFfYdH8Gipec8Eeeu0xXdbba9frFj0=OqFfea0dXdd9vqai=hGuQ8kuc9pgc9s8qqaq=dirpe0xb9q8qiLsFr0=vr0=vr0dc8meaabaqaciaacaGaaeqabaqabeGadaaakeaaieWacuWF2bGDgaqeamaaBaaaleaacqWFQbGAaeqaaOGaeiikaGIae8hBaW2aaSbaaSqaaiabigdaXaqabaGccqGGSaalcqGGUaGlcqGGUaGlcqGGUaGlcqGGSaalcqWFSbaBdaWgaaWcbaGae8NBa42aaSbaaWqaaiab=bhaWjab=9gaVjab=nhaZbqabaaaleqaaOGaeiykaKcaaa@3FD0@ and the learning set mean value:

Tj=−Φ(v¯j(l1,...,lnpos)−v¯jLS(l1,...,lnpos))22σjLS(l1,...,lnpos)2     (11)
 MathType@MTEF@5@5@+=feaafiart1ev1aaatCvAUfKttLearuWrP9MDH5MBPbIqV92AaeXatLxBI9gBaebbnrfifHhDYfgasaacH8akY=wiFfYdH8Gipec8Eeeu0xXdbba9frFj0=OqFfea0dXdd9vqai=hGuQ8kuc9pgc9s8qqaq=dirpe0xb9q8qiLsFr0=vr0=vr0dc8meaabaqaciaacaGaaeqabaqabeGadaaakeaaieWacqWFubavdaWgaaWcbaGae8NAaOgabeaakiabg2da9iabgkHiTiabfA6agnaalaaabaWaaeWaaeaacuWF2bGDgaqeamaaBaaaleaacqWFQbGAaeqaaOWaaeWaaeaacqWFSbaBdaWgaaWcbaGaeGymaedabeaakiabcYcaSiabc6caUiabc6caUiabc6caUiabcYcaSiab=XgaSnaaBaaaleaacqWFUbGBdaWgaaadbaGae8hCaaNae83Ba8Mae83CamhabeaaaSqabaaakiaawIcacaGLPaaacqGHsislcuWF2bGDgaqeamaaBaaaleaacqWFQbGAdaWgaaadbaGae8htaWKae83uamfabeaaaSqabaGcdaqadaqaaiab=XgaSnaaBaaaleaacqaIXaqmaeqaaOGaeiilaWIaeiOla4IaeiOla4IaeiOla4IaeiilaWIae8hBaW2aaSbaaSqaaiab=5gaUnaaBaaameaacqWFWbaCcqWFVbWBcqWFZbWCaeqaaaWcbeaaaOGaayjkaiaawMcaaaGaayjkaiaawMcaamaaCaaaleqabaGaeGOmaidaaaGcbaGaeGOmaidcciGae43Wdm3aaSbaaSqaaiab=PgaQnaaBaaameaacqWFmbatcqWFtbWuaeqaaaWcbeaakmaabmaabaGae8hBaW2aaSbaaSqaaiabigdaXaqabaGccqGGSaalcqGGUaGlcqGGUaGlcqGGUaGlcqGGSaalcqWFSbaBdaWgaaWcbaGae8NBa42aaSbaaWqaaiab=bhaWjab=9gaVjab=nhaZbqabaaaleqaaaGccaGLOaGaayzkaaWaaWbaaSqabeaacqaIYaGmaaaaaOGaaCzcaiaaxMaadaqadaqaaiabigdaXiabigdaXaGaayjkaiaawMcaaaaa@7B65@

The criterion Φ_*j *_in equation 11 determines whether a penalty is applied or not. Depending on whether small or great property values should be penalized, Φ_*j *_can be expressed using the two equations below. Here, the potentially freely selectable parameter ***t***_*j *_≥ 0 can be used to change the stringency of the threshold.

Φj={1ifv¯j(l1,...,lnpos)<v¯jLS(l1,...,lnpos)−tjσj(l1,...,lnpos)0ifv¯j(l1,...,lnpos)≥v¯jLS(l1,...,lnpos)−tjσj(l1,...,lnpos)     (12)
 MathType@MTEF@5@5@+=feaafiart1ev1aaatCvAUfKttLearuWrP9MDH5MBPbIqV92AaeXatLxBI9gBaebbnrfifHhDYfgasaacH8akY=wiFfYdH8Gipec8Eeeu0xXdbba9frFj0=OqFfea0dXdd9vqai=hGuQ8kuc9pgc9s8qqaq=dirpe0xb9q8qiLsFr0=vr0=vr0dc8meaabaqaciaacaGaaeqabaqabeGadaaakeaacqqHMoGrdaWgaaWcbaacbmGae8NAaOgabeaakiabg2da9maaceqabaqbaeqabiWaaaqaaiabigdaXaqaaiab=LgaPjab=zgaMbqaaiqb=zha2zaaraWaaSbaaSqaaiab=PgaQbqabaGcdaqadaqaaiab=XgaSnaaBaaaleaacqaIXaqmaeqaaOGaeiilaWIaeiOla4IaeiOla4IaeiOla4IaeiilaWIae8hBaW2aaSbaaSqaaiab=5gaUnaaBaaameaacqWFWbaCcqWFVbWBcqWFZbWCaeqaaaWcbeaaaOGaayjkaiaawMcaaiabgYda8iqb=zha2zaaraWaaSbaaSqaaiab=PgaQnaaBaaameaacqWFmbatcqWFtbWuaeqaaaWcbeaakmaabmaabaGae8hBaW2aaSbaaSqaaiabigdaXaqabaGccqGGSaalcqGGUaGlcqGGUaGlcqGGUaGlcqGGSaalcqWFSbaBdaWgaaWcbaGae8NBa42aaSbaaWqaaiab=bhaWjab=9gaVjab=nhaZbqabaaaleqaaaGccaGLOaGaayzkaaGaeyOeI0Iae8hDaq3aaSbaaSqaaiab=PgaQbqabaacciGccqGFdpWCdaWgaaWcbaGae8NAaOgabeaakmaabmaabaGae8hBaW2aaSbaaSqaaiabigdaXaqabaGccqGGSaalcqGGUaGlcqGGUaGlcqGGUaGlcqGGSaalcqWFSbaBdaWgaaWcbaGae8NBa42aaSbaaWqaaiab=bhaWjab=9gaVjab=nhaZbqabaaaleqaaaGccaGLOaGaayzkaaaabaGaeGimaadabaGae8xAaKMae8NzaygabaGaf8NDayNbaebadaWgaaWcbaGae8NAaOgabeaakmaabmaabaGae8hBaW2aaSbaaSqaaiabigdaXaqabaGccqGGSaalcqGGUaGlcqGGUaGlcqGGUaGlcqGGSaalcqWFSbaBdaWgaaWcbaGae8NBa42aaSbaaWqaaiab=bhaWjab=9gaVjab=nhaZbqabaaaleqaaaGccaGLOaGaayzkaaGaeyyzImRaf8NDayNbaebadaWgaaWcbaGae8NAaO2aaSbaaWqaaiab=Xeamjab=nfatbqabaaaleqaaOWaaeWaaeaacqWFSbaBdaWgaaWcbaGaeGymaedabeaakiabcYcaSiabc6caUiabc6caUiabc6caUiabcYcaSiab=XgaSnaaBaaaleaacqWFUbGBdaWgaaadbaGae8hCaaNae83Ba8Mae83CamhabeaaaSqabaaakiaawIcacaGLPaaacqGHsislcqWF0baDdaWgaaWcbaGae8NAaOgabeaakiab+n8aZnaaBaaaleaacqWFQbGAaeqaaOWaaeWaaeaacqWFSbaBdaWgaaWcbaGaeGymaedabeaakiabcYcaSiabc6caUiabc6caUiabc6caUiabcYcaSiab=XgaSnaaBaaaleaacqWFUbGBdaWgaaadbaGae8hCaaNae83Ba8Mae83CamhabeaaaSqabaaakiaawIcacaGLPaaaaaGaaCzcaiaaxMaadaqadaqaaiabigdaXiabikdaYaGaayjkaiaawMcaaaGaay5Eaaaaaa@BF7C@

Φj={1ifv¯j(l1,...,lnpos)>v¯jLS(l1,...,lnpos)+tjσj(l1,...,lnpos)0ifv¯j(l1,...,lnpos)≤v¯jLS(l1,...,lnpos)+tjσj(l1,...,lnpos)     (13)
 MathType@MTEF@5@5@+=feaafiart1ev1aaatCvAUfKttLearuWrP9MDH5MBPbIqV92AaeXatLxBI9gBaebbnrfifHhDYfgasaacH8akY=wiFfYdH8Gipec8Eeeu0xXdbba9frFj0=OqFfea0dXdd9vqai=hGuQ8kuc9pgc9s8qqaq=dirpe0xb9q8qiLsFr0=vr0=vr0dc8meaabaqaciaacaGaaeqabaqabeGadaaakeaacqqHMoGrdaWgaaWcbaacbmGae8NAaOgabeaakiabg2da9maaceqabaqbaeqabiWaaaqaaiabigdaXaqaaiab=LgaPjab=zgaMbqaaiqb=zha2zaaraWaaSbaaSqaaiab=PgaQbqabaGcdaqadaqaaiab=XgaSnaaBaaaleaacqaIXaqmaeqaaOGaeiilaWIaeiOla4IaeiOla4IaeiOla4IaeiilaWIae8hBaW2aaSbaaSqaaiab=5gaUnaaBaaameaacqWFWbaCcqWFVbWBcqWFZbWCaeqaaaWcbeaaaOGaayjkaiaawMcaaiab=5da+iqb=zha2zaaraWaaSbaaSqaaiab=PgaQnaaBaaameaacqWFmbatcqWFtbWuaeqaaaWcbeaakmaabmaabaGae8hBaW2aaSbaaSqaaiabigdaXaqabaGccqGGSaalcqGGUaGlcqGGUaGlcqGGUaGlcqGGSaalcqWFSbaBdaWgaaWcbaGae8NBa42aaSbaaWqaaiab=bhaWjab=9gaVjab=nhaZbqabaaaleqaaaGccaGLOaGaayzkaaGaey4kaSIae8hDaq3aaSbaaSqaaiab=PgaQbqabaacciGccqGFdpWCdaWgaaWcbaGae8NAaOgabeaakmaabmaabaGae8hBaW2aaSbaaSqaaiabigdaXaqabaGccqGGSaalcqGGUaGlcqGGUaGlcqGGUaGlcqGGSaalcqWFSbaBdaWgaaWcbaGae8NBa42aaSbaaWqaaiab=bhaWjab=9gaVjab=nhaZbqabaaaleqaaaGccaGLOaGaayzkaaaabaGaeGimaadabaGae8xAaKMae8NzaygabaGaf8NDayNbaebadaWgaaWcbaGae8NAaOgabeaakmaabmaabaGae8hBaW2aaSbaaSqaaiabigdaXaqabaGccqGGSaalcqGGUaGlcqGGUaGlcqGGUaGlcqGGSaalcqWFSbaBdaWgaaWcbaGae8NBa42aaSbaaWqaaiab=bhaWjab=9gaVjab=nhaZbqabaaaleqaaaGccaGLOaGaayzkaaGaeyizImQaf8NDayNbaebadaWgaaWcbaGae8NAaO2aaSbaaWqaaiab=Xeamjab=nfatbqabaaaleqaaOWaaeWaaeaacqWFSbaBdaWgaaWcbaGaeGymaedabeaakiabcYcaSiabc6caUiabc6caUiabc6caUiabcYcaSiab=XgaSnaaBaaaleaacqWFUbGBdaWgaaadbaGae8hCaaNae83Ba8Mae83CamhabeaaaSqabaaakiaawIcacaGLPaaacqGHRaWkcqWF0baDdaWgaaWcbaGae8NAaOgabeaakiab+n8aZnaaBaaaleaacqWFQbGAaeqaaOWaaeWaaeaacqWFSbaBdaWgaaWcbaGaeGymaedabeaakiabcYcaSiabc6caUiabc6caUiabc6caUiabcYcaSiab=XgaSnaaBaaaleaacqWFUbGBdaWgaaadbaGae8hCaaNae83Ba8Mae83CamhabeaaaSqabaaakiaawIcacaGLPaaaaaGaaCzcaiaaxMaadaqadaqaaiabigdaXiabiodaZaGaayjkaiaawMcaaaGaay5Eaaaaaa@BF54@

In our concept, the value ***t***_*j *_is not thought to be an adjustable parameter depending on the learning set. Generally, the value ***t***_*j *_is set equal to zero for all Gaussian-type terms but needs to be determined for the fixed penalties to define the level of the threshold (see Table 6). We see the introduction of ***t***_*j *_as a way to achieve equal formal notation of Gaussian terms and fixed penalties.

**Table 6:** Summary of the physical terms *T*_*j *_in the scoring function of the pkaPS predictor.

*T*_*j*_	Property	Positions	α_ppt,j_	Description
*T*_1_	(+) H, K, R	-3/-2	1.0	Positive charge
*T*_2_	EISD860102 [29]	-3/-2	0.030	Hydrophilic residues
*T*_3_	ZIMJ680104 [87]	-6 to -2	0.020	Isoelectric point (positive charge), long range
*T*_4_	(+) H, K, R; (-) D, E	-6 to -2	0.48	Total charge, long range
*T*_5_	GEIM800106 [34]	+1	0.070	β-strand preference
*T*_6_	GEIM800107 [34]	+1/+4	0.040	β-strand preference, compensated
*T*_7_	HAGECH94_V [88]	+2/+3	0.040	Size restrictions
*T*_8_	KARP850101 [89]	+3	0.040	Flexibility
*T*_9_	KARP850101 [89]	-9 to -4	0.040	Minimal linker – flexibility
*T*_10_	EISD840101 [29]	-9 to -4	0.040	Minimal linker – hydrophilicity
*T*_11_	EISD840101 [29]	+4 to +9	0.058	Minimal linker – hydrophilicity
*T*_12_	KARP850101 [89]	+4 to +9	0.058	Minimal linker – flexibility
*T*_13_	CIDH920105 [90]	-18 to -6, +6 to +23	0.040	Avoid buried regions – hydrophilicity
*T*_14_	VINM940101 [30]	-18 to -6, +6 to +23	0.040	Avoid buried regions – flexibility

The table shows the complete list of physical property terms in the score function. The values ***t***_*j *_in equations 12 and 13 are set equal to zero for Gaussian-type terms and equal to 0.1 for fixed penalties ***T***_1 _and ***T***_4_. The only adjustable parameter per term is the weight α_ppt,j _(equation 10). These parameters have been selected so that ***S***_ppt _is close to zero for most of the learning set examples. The values *α*_*profile,j *_(equation 9) for the positions -6...+6 are the following multiples of a normalization factor 0.051: 7, 6, 3, 5, 6, 6, 6, 3, 2, 5, 3, 4, and 1. Initial guesses for the *α*_ppt,j _and *α*_profile,j _parameters have been calculated with linear kernel support vector machines as implemented in the LIBSVM library [91]. These weights have subsequently been rounded to two significant positions and edited manually to avoid non-positive numbers and to achieve close-to-zero ***T***_*j *_values for the learning set sequences.

### Evaluation of predictor performance

The prediction outcomes of an algorithm can be grouped into the following four categories: "true-positives (*T*_*P*_)" are correctly predicted queries that contain the analyzed feature; "false-negatives (*F*_*N*_)" contain the feature but are predicted not to do so; "true-negatives (*T*_*N*_)" are correctly predicted not to contain the feature; "false-positives (*F*_*P*_)" do not contain the feature but are wrongly predicted to do so. The number of prediction results that fall into these categories are used to calculate measures for predictor performances. These are typically calculated in terms of sensitivity (*S*_*n*_) and specificity (*S*_*p*_). The former is defined as the proportion of positive sites that the method can identify, and the latter as the fraction of negative sites that is correctly classified [12,78].

Sn=TPTP+FN     (14)
 MathType@MTEF@5@5@+=feaafiart1ev1aaatCvAUfKttLearuWrP9MDH5MBPbIqV92AaeXatLxBI9gBaebbnrfifHhDYfgasaacH8akY=wiFfYdH8Gipec8Eeeu0xXdbba9frFj0=OqFfea0dXdd9vqai=hGuQ8kuc9pgc9s8qqaq=dirpe0xb9q8qiLsFr0=vr0=vr0dc8meaabaqaciaacaGaaeqabaqabeGadaaakeaaieWacqWFtbWudaWgaaWcbaGae8NBa4gabeaakiabg2da9maalaaabaGae8hvaq1aaSbaaSqaaiab=bfaqbqabaaakeaacqWFubavdaWgaaWcbaGae8huaafabeaakiabgUcaRiab=zeagnaaBaaaleaacqWFobGtaeqaaaaakiaaxMaacaWLjaWaaeWaaeaacqaIXaqmcqaI0aanaiaawIcacaGLPaaaaaa@3D9D@

Sp=TNFP+TN     (15)
 MathType@MTEF@5@5@+=feaafiart1ev1aaatCvAUfKttLearuWrP9MDH5MBPbIqV92AaeXatLxBI9gBaebbnrfifHhDYfgasaacH8akY=wiFfYdH8Gipec8Eeeu0xXdbba9frFj0=OqFfea0dXdd9vqai=hGuQ8kuc9pgc9s8qqaq=dirpe0xb9q8qiLsFr0=vr0=vr0dc8meaabaqaciaacaGaaeqabaqabeGadaaakeaaieWacqWFtbWudaWgaaWcbaGae8hCaahabeaakiabg2da9maalaaabaGae8hvaq1aaSbaaSqaaiab=5eaobqabaaakeaacqWFgbGrdaWgaaWcbaGae8huaafabeaakiabgUcaRiab=rfaunaaBaaaleaacqWFobGtaeqaaaaakiaaxMaacaWLjaWaaeWaaeaacqaIXaqmcqaI1aqnaiaawIcacaGLPaaaaaa@3D9F@

Alternatively, one can use the "false-negative" (*F*_*n*_) and "false-positive" (*F*_*p*_) rates. They express the opposite of sensitivity and specificity, namely the amount of wrongly classified sequences for each prediction class, and are equal to 1 minus the respective *S*_*n *_or *S*_*p *_values.

### "On the fly" estimation of false-positive rates

To assess false-positive rates "on the fly" for obtained total scores *S*, a previously described estimation methodology [17,20,79,80] is used that follows the spirit of BLAST *p*-values [81]. This allows an easier interpretation of the total score *S *and provides the possibility for a better comparison with outputs from other prediction programs. The probability of false-positive prediction is approximated to the empirical distribution of sequences that are known not to carry the feature of interest. If a set of negative examples exists, it can be directly used for this task. If none is available, the function can be extrapolated from the distribution of low scores.

The generalized analytical form of the extreme-value distribution that has successfully been applied in the MyPS [20] and big-Π predictors [17,79] is used for this approximation task. The probability *P *of a score *S *to be larger than a threshold *S*_*th *_is calculated using a polynomial ***f ***(***S***_***th***_) of the score threshold *S*_*th *_and can be described by:

P(S>Sth)=1−e−e−f(Sth)     (16)
 MathType@MTEF@5@5@+=feaafiart1ev1aaatCvAUfKttLearuWrP9MDH5MBPbIqV92AaeXatLxBI9gBaebbnrfifHhDYfgasaacH8akY=wiFfYdH8Gipec8Eeeu0xXdbba9frFj0=OqFfea0dXdd9vqai=hGuQ8kuc9pgc9s8qqaq=dirpe0xb9q8qiLsFr0=vr0=vr0dc8meaabaqaciaacaGaaeqabaqabeGadaaakeaaieWacqWFqbaucqGGOaakcqWFtbWucqGH+aGpcqWFtbWudaWgaaWcbaGae8hDaqNae8hAaGgabeaakiabcMcaPiabg2da9iabigdaXiabgkHiTiab=vgaLnaaCaaaleqabaGaeyOeI0Iae8xzau2aaWbaaWqabeaacqGHsislcqWFMbGzcqGGOaakcqWFtbWudaWgaaqaaiab=rha0jab=HgaObqabaGaeiykaKcaaaaakiaaxMaacaWLjaWaaeWaaeaacqaIXaqmcqaI2aGnaiaawIcacaGLPaaaaaa@496D@

where

f(Sth)=∑i=1nλi(Sth−u)i     (17)
 MathType@MTEF@5@5@+=feaafiart1ev1aaatCvAUfKttLearuWrP9MDH5MBPbIqV92AaeXatLxBI9gBaebbnrfifHhDYfgasaacH8akY=wiFfYdH8Gipec8Eeeu0xXdbba9frFj0=OqFfea0dXdd9vqai=hGuQ8kuc9pgc9s8qqaq=dirpe0xb9q8qiLsFr0=vr0=vr0dc8meaabaqaciaacaGaaeqabaqabeGadaaakeaaieWacqWFMbGzcqGGOaakcqWFtbWudaWgaaWcbaGae8hDaqNae8hAaGgabeaakiabcMcaPiabg2da9maaqahabaacciGae43UdW2aaSbaaSqaaiab=LgaPbqabaaabaGae8xAaKMaeyypa0JaeGymaedabaGae8NBa4ganiabggHiLdGcdaqadaqaaiab=nfatnaaBaaaleaacqWF0baDcqWFObaAaeqaaOGaeyOeI0Iae8xDauhacaGLOaGaayzkaaWaaWbaaSqabeaacqWFPbqAaaGccaWLjaGaaCzcamaabmaabaGaeGymaeJaeG4naCdacaGLOaGaayzkaaaaaa@4D5F@

The qualities of the fits are evaluated with the residual *R*_*n *_of the least-squares fit for all sequences *k *included in each fit evaluation (1 ≤ *k *≤ *n*_*seq*_; *n*_*seq *_is the number of sequences included in fit evaluation, *S*_*th*,*k *_is the total score for the *k*^th ^sequence):

Rn=∑j=1nseq〈−ln⁡{−ln⁡[1−Pempirical(S<Sth,k)]}−f(Sth,k)〉2     (18)
 MathType@MTEF@5@5@+=feaafiart1ev1aaatCvAUfKttLearuWrP9MDH5MBPbIqV92AaeXatLxBI9gBaebbnrfifHhDYfgasaacH8akY=wiFfYdH8Gipec8Eeeu0xXdbba9frFj0=OqFfea0dXdd9vqai=hGuQ8kuc9pgc9s8qqaq=dirpe0xb9q8qiLsFr0=vr0=vr0dc8meaabaqaciaacaGaaeqabaqabeGadaaakeaaieWacqWFsbGudaWgaaWcbaGae8NBa4gabeaakiabg2da9maaqahabaWaaaWabeaacqGHsislcyGGSbaBcqGGUbGBdaGadeqaaiabgkHiTiGbcYgaSjabc6gaUnaadmaabaGaeGymaeJaeyOeI0Iae8huaa1aaSbaaSqaaiab=vgaLjab=1gaTjab=bhaWjab=LgaPjab=jhaYjab=LgaPjab=ngaJjab=fgaHjab=XgaSbqabaGcdaqadaqaaiab=nfatjabgYda8iab=nfatnaaBaaaleaacqWF0baDcqWFObaAcqGGSaalcqWFRbWAaeqaaaGccaGLOaGaayzkaaaacaGLBbGaayzxaaaacaGL7bGaayzFaaGaeyOeI0Iae8Nzay2aaeWaaeaacqWFtbWudaWgaaWcbaGae8hDaqNae8hAaGMaeiilaWIae83AaSgabeaaaOGaayjkaiaawMcaaaGaayzkJiaawQYiamaaCaaaleqabaGaeGOmaidaaOGaaCzcaiaaxMaadaqadaqaaiabigdaXiabiIda4aGaayjkaiaawMcaaaWcbaGae8NAaOMaeyypa0JaeGymaedabaGae8NBa42aaSbaaWqaaiab=nhaZjab=vgaLjab=fhaXbqabaaaniabggHiLdaaaa@7289@

Approximations of the empirical distributions are calculated using iterative non-linear curve fitting implemented in the XMGRACE tool [82].

### Predictor implementation

The predictor for protein kinase A (PKA) dependent phosphorylation, pkaPS, integrates the motif-related knowledge presented in the preceding sections. The profile term S_profile _is calculated using positions -6 to +6. The implemented terms *T*_*j *_reflect the structure of the substrate motif as deduced from the available sequence, structural and kinetic data. The main determinants for substrate specificity are the residues that interact with the enzyme in its binding pocket, and the adjacent positions at the mouth of the cavity. Various terms analyze these amino acids and combinations thereof for deviations from the typical physico-chemical motif fingerprint. Another group of terms evaluates the quality of the linkers that flank this region. These must have a minimal length to ensure that the phosphorylation site and its adjacent positions are sufficiently separated from the core of the respective substrate protein. The last set of terms is calculated over a region that extends further than the minimal linker length. The purpose of these functions is to exclude hydrophobic domains that might fold to protein cores, and thereby become inaccessible for substrate recognition. A summary of these terms, including the utilized physico-chemical properties, the implicated positions and references to the underlying rationales is presented in Table 6.

### False-positive prediction rate within sets of proven negative examples

**Figure 8:**
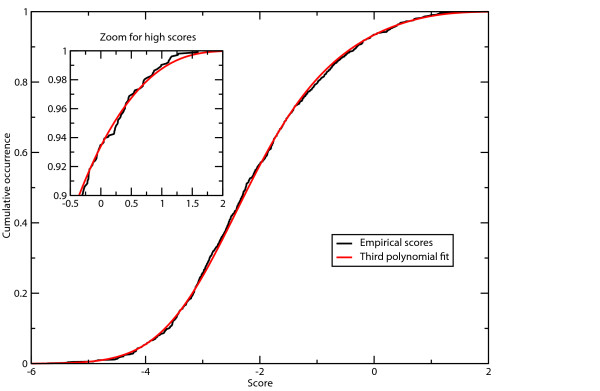
**Approximation of the empirical score distribution of non-phosphorylated sites**. The empirical score distribution was approximated using equations 16 and 17. With a correlation coefficient of 0.9988, the applied polynomial fit of 3^rd ^order provides a sufficiently accurate approximation of the expected false-positive rate. The parameters with respect to equation 17 are: *u *= -1.76847, λ_1 _= -0.766775, λ_2 _= 0.166677 and λ_3 _= -0.0298602. The polynomial fit was calculated using the XMGRACE tool [81].

The 1026 unphosphorylated serines/threonines that were collected in the course of learning set construction served as a basis to evaluate the false-positive prediction rate of the pkaPS tool. A program run over these sequences revealed that 6.5% of the included entries produced scores *S *≥ 0, and, thus, can be classified as false-positives. To assess the false-positive rate for any produced score *S *on the fly, an analytical score distribution was generated using the methodology presented above. Due to the availability of a real set of non-phosphorylated sequences, the analytical distribution could directly be approximated to the empirical score distribution of the set of negative examples (Figure 8).

## Reviewers' comments

### Reviewer's report I

Erik van Nimwegen, Biozentrum, University of Basel, Switzerland.

This is a very thorough description of an algorithm for identifying phosphorylation sites of Protein Kinase A (PKA). It is clear that the authors put a lot of effort in deciding which physical features to use and how to use them. I am generally quite convinced that the pkaPS predictor provides the current state-of-the-art for PKA phosporylation site prediction. Therefore this is clearly a very worthwhile paper. I have two main points of criticism:

• The paper is too long. I appreciate all the information that the authors provide but I think the paper could be made much more readable by moving a lot of the material to supplementary materials and leaving a much more condensed and structured description of the key points. Right now there is just too much material to wade through for the reader to get a good overview of what is being done.

Author response: *Our previous predictor developments have always been described in a pair of papers – one for the analysis of the property pattern near the modification site, another for the description and validation of the predictor. Thus, two different but related scientific tasks have been composed into one text. Further, we wish to supply all information that an interested reader can recreate the whole work and the implementation of the predictor. We feel that none of the information provided is dispensable. Nevertheless, we understand the concerns of the reviewer and decided to add an introductory overview section to the Results that summarizes the purpose of the respective sections and the major results described therein*.

• I have concerns about over-fitting. There are a lot of parameters that go into the method that seem to have been set by hand (actually it is not entirely clear from the text how the parameters were set. This could be better explained). Examples are the collections of *α *weights and the thresholds *t*_*j *_. Given this moderately large set of parameters that have been tuned by the authors one wonders about over-fitting. In the description of the neighbor-jackknife test there is mention of "the parameterization procedure (neighbor-jackknife test, Materials and Methods)" but I did not see any description of this parameterization procedure. To address over-fitting, I propose that the authors do something like randomly dividing both the data set of positive examples as well as the set of negative examples in half. The parameters of the model should then be tuned independently on these two half-sets and false positive/negative rates can then be estimated by applying the two predictors to the half-sets not used in the training.

Author response: *The revised version of the Methods section clarifies that the values ****t***_***j ***_*have not been used as adjustable parameters but as a concept to formally unify Gaussian-type physical property terms and fixed penalties. The values ****t***_***j ***_*have been described in the legend of Table *6*. The only adjustable parameters in the physical property term part of the score are the 14 α*_***ppt,j***_*. These are listed in *Table 6* and the procedure for their determination is specified in the legend of Table *6*. The exact values of the α*_***ppt,j***_* are not critical since the physical property terms never generate a positive score (their purpose is to penalize non-permissive queries) and it is only important that the physical property terms do generate values close to zero for most of the learning set sequences. In the initial versions of the score function, each physical property term was even checked individually against the learning set to find maximal values for the α*_***ppt,j***_*. Simple linear kernel support vector machines were used to obtain optimized guesses and, thus, to reduce the size of the twilight zone, a zone of scores indicating unclear hits. The question of parameter overfitting has already been answered in the neighbor jack-knife test when whole homologous groups of sequences have been taken out of the learning set. This is a more rigorous approach compared with the random selection of sequences since the score function can be biased already due to the occurrence of a single homologue in the set*.

• page 3: "a prototypic model for the kinase group". In what sense is PKA prototypic?

Author response:*The reformulation emphasizes that PKA is the one of the best studied kinases and, therefore, well suited for substrate site predictor development*.

• page 30: "The fact that phosphorylation frequently occurs .... is shown in table 4." I don't understand the reasoning here. In table 5 the number of phosphorylation sites per protein just seems to fall exponentially suggesting that the distribution might simple be a Poisson distribution, i.e no particular bias toward having multiple sites per protein.

Author response:*The respective paragraph has been expanded to clarify that we just wish to describe the phosphorylation site distribution of the learning set. We do not intend to postulate a specific bias except for the observation that, if multiple sites do occur in one protein, they tend to cluster together (see Results)*.

• Page 33: Quantity *R*(*l*). Is this quantity ever used in the predictor? If not, what is the use of introducing it here?

Author response: *The equations described in the Methods section "Sequence analysis part 2. Derivation of physical property characteristics" are used to filter the physical property pattern (see first three sections of the Results) prior to predictor development. We added text to this section to clarify this issue*.

• page 35: "using the PSIC algorithm..." I am confused because on page 31 it was mentioned that the "sum of mismatches" method of Vingron and Argos is used.

Author response:*Redundancy removal due to the occurrence of homologous sequences in the learning set is carried out differently for the physical property terms (with a modification of the Vingron-Argos procedure *[74,75]*) and for the profile term (with the PSIC method *[77]*). The PSIC method is more sensitive but requires independent consideration of alignment positions. In physical property terms, we regularly consider multiple positions and the PSIC concept is formally not applicable in this context*.

• page 39: "*S*_*th *_is a polynomial..." The expression in (16) is not a polynomial.

Author response:*True*, ***f***(***S***_***th***_)* is the polynomial function considered here. We reformulated the respective part*.

### Reviewer's report II

Sandor Pongor, International Centre for Genetic Engineering and Biotechnology, Trieste, Italy.

The manuscript of Neuberger et al "pkaPS: Prediction of Protein Kinase A Phosphorylation Sites with the Simplified Kinase-Substrate Binding Model" describes an heuristic method for describing PKA phosphorylation sites based on the distribution of various physicochemical parameters in the region flanking the phosphorylated residue as well as information on the foldedness of the polypeptide region. They present a scoring function that can confidently discriminate PKA phosphorylation sites from S/T residues in other environments. The predictor is made publicly available on a website. The description of the work is detailed and reproducible, and is in line with the groups previous works on similar subjects. The improvement over the other existing methods is convincing, and the idea of combining a physically reasonable model with statistical learning is an attractive one. I suggest the manuscript be published without modifications.

### Reviewer's report III

Igor Zhulin, University of Tennessee, Oak Ridge National Laboratory, USA.

In this paper, authors present the development of a prediction tool termed "pkaPS" for the purpose of identifying substrate proteins for the serine/threonine kinase PKA. Through a very thorough sequence/structure analysis, authors built a PKA-specific binding motif model, which can discriminate between PKA phosphorylation sites and other potential serine/threonine sites.

In my opinion, both the strength and the weakness of this paper are in its very detailed format. The manuscript is quite long and jam-packed with information even though the authors moved most technical details into the methods section. I am sure that bioinformaticians will find this paper very interesting, whereas most biologists are unlikely to reach the third page of the results section. This is unfortunate, because some of the derived predictions would be quite interesting for them (see below). I recommend adding information on biologically relevant predictions to the abstract at the expense of some technical details. This may capture attention of those to whom this information is addressed.

While skipping some details, I managed to follow authors' logic, which eventually resulted in building the analytical model for the kinase binding motif. I have to admit that this is a very difficult and noble task. The tendency to produce large numbers of false positives is a signature of most "sequence-only" motif predictors, and any attempt to overcome this problem inevitably leads to the need to incorporate chemistry and structure into the model. The authors did just that.

Ultimately, the success of the new predictive method and associated tool will be measured by the number of correctly identified targets (although shown measures of sensitivity and specificity are important). Authors indicate that when applied to the human proteome, the predictor ranked most highly protein families that are known PKA targets, such as histone H2A. I found it very intriguing that the top scorers also include ribosomal proteins L21e, L22, L6 that are not known to undergo phosphorylation or interact with protein kinases. However, there is a new body of evidence that some ribosomal proteins, for example S6, can be phosphorylated by specific protein kinases (Ruvinsky & Meyuhas, 2006 Trends Biochem Sci 31:342-8). Thus, predictions look very exciting and indeed produce testable hypotheses that might lead to novel discoveries in eukaryotic signal transduction.

Author response: *Similar to the first reviewer, this referee expresses his concern with respect to readability of the article. We think that the new introductory overview section of the Results removes these concerns. We are grateful for the hint to the Ruvinsky & Meyuhas article that supports some of the predictions in this work. We complement the summary with some of our biological results*.

## Availability and requirements

The prediction tool is available as WWW server at  and it is thought for fair use. Please contact the authors if large sets (>500 sequences) are planned to be analyzed. The access is possible with any web-browsing tool.

## Competing interest

The authors declare that they have no competing interests.

## Authors' contributions

GN and FE designed the study and evaluated the results. GN and GS carried out the programming and the sequence analytic work. All authors participated in drafting the manuscript and approved the final version.

## References

[B1] Madhani H (2006). Functional analysis of protein kinase networks in living cells: beyond "knock-outs" and "knock-downs". Methods.

[B2] Knighton DR, Zheng JH, Teneyck LF, Ashford VA, Xuong NH, Taylor SS, Sowadski JM (1991). Crystal-Structure of the Catalytic Subunit of Cyclic Adenosine-Monophosphate Dependent Protein-Kinase. Science.

[B3] Knighton DR, Zheng JH, Teneyck LF, Xuong NH, Taylor SS, Sowadski JM (1991). Structure of A Peptide Inhibitor Bound to the Catalytic Subunit of Cyclic Adenosine-Monophosphate Dependent Protein-Kinase. Science.

[B4] Bramson HN, Kaiser ET, Mildvan AS (1984). Mechanistic Studies of Camp-Dependent Protein-Kinase Action. Crc Critical Reviews in Biochemistry.

[B5] Kemp BE, Graves DJ, Benjamini E, Krebs EG (1977). Role of Multiple Basic Residues in Determining Substrate-Specificity of Cyclic Amp-Dependent Protein-Kinase. Journal of Biological Chemistry.

[B6] Walsh DA, Vanpatten SM (1994). Multiple Pathway Signal-Transduction by the Camp-Dependent Protein-Kinase. Faseb Journal.

[B7] Moore MJ, Adams JA, Taylor SS (2003). Structural basis for peptide binding in protein kinase A - Role of glutamic acid 203 and tyrosine 204 in the peptide-positioning loop. Journal of Biological Chemistry.

[B8] Blom N, Gammeltoft S, Brunak S (1999). Sequence and structure-based prediction of eukaryotic protein phosphorylation sites. Journal of Molecular Biology.

[B9] Falquet L, Pagni M, Bucher P, Hulo N, Sigrist CJA, Hofmann K, Bairoch A (2002). The PROSITE database, its status in 2002. Nucleic Acids Research.

[B10] Sigrist CJ, Cerutti L, Hulo N, Gattiker A, Falquet L, Pagni M, Bairoch A, Bucher P (2002). PROSITE: a documented database using patterns and profiles as motif descriptors. Brief Bioinform.

[B11] Hulo N, Sigrist CJ, Le S, Langendijk-Genevaux PS, Bordoli L, Gattiker A, De Castro E, Bucher P, Bairoch A (2004). Recent improvements to the PROSITE database. Nucleic Acids Res.

[B12] Blom N, Sicheritz-Ponten T, Gupta R, Gammeltoft S, Brunak S (2004). Prediction of post-translational glycosylation and phosphorylation of proteins from the amino acid sequence. Proteomics.

[B13] Obenauer JC, Cantley LC, Yaffe MB (2003). Scansite 2.0: Proteome-wide prediction of cell signaling interactions using short sequence motifs. Nucleic Acids Res.

[B14] Kim JH, Lee J, Oh B, Kimm K, Koh I (2004). Prediction of phosphorylation sites using SVMs. Bioinformatics.

[B15] Zhou FF, Xue Y, Chen GL, Yao X (2004). GPS: a novel group-based phosphorylation predicting and scoring method. Biochem Biophys Res Commun.

[B16] Xue Y, Zhou F, Zhu M, Ahmed K, Chen G, Yao X (2005). GPS: a comprehensive www server for phosphorylation sites prediction. Nucleic Acids Res.

[B17] Eisenhaber B, Bork P, Eisenhaber F (1999). Prediction of potential GPI-modification sites in proprotein sequences. J Mol Biol.

[B18] Eisenhaber B, Wildpaner M, Schultz CJ, Borner GH, Dupree P, Eisenhaber F (2003). Glycosylphosphatidylinositol lipid anchoring of plant proteins. Sensitive prediction from sequence- and genome-wide studies for Arabidopsis and rice. Plant Physiol.

[B19] Eisenhaber B, Schneider G, Wildpaner M, Eisenhaber F (2004). A sensitive predictor for potential GPI lipid modification sites in fungal protein sequences and its application to genome-wide studies for Aspergillus nidulans, Candida albicans, Neurospora crassa, Saccharomyces cerevisiae and Schizosaccharomyces pombe. J Mol Biol.

[B20] Maurer-Stroh S, Eisenhaber B, Eisenhaber F (2002). N-terminal N-myristoylation of proteins: prediction of substrate proteins from amino acid sequence. J Mol Biol.

[B21] Maurer-Stroh S, Eisenhaber F (2005). Refinement and prediction of protein prenylation motifs. Genome Biol.

[B22] Neuberger G, Maurer-Stroh S, Eisenhaber B, Hartig A, Eisenhaber F (2003). Prediction of peroxisomal targeting signal 1 containing proteins from amino acid sequence. J Mol Biol.

[B23] Eisenhaber B, Bork P, Eisenhaber F (1998). Sequence properties of GPI-anchored proteins near the omega-site: constraints for the polypeptide binding site of the putative transamidase. Protein Eng.

[B24] Neuberger G, Maurer-Stroh S, Eisenhaber B, Hartig A, Eisenhaber F (2003). Motif refinement of the peroxisomal targeting signal 1 and evaluation of taxon-specific differences. J Mol Biol.

[B25] Songyang Z, Blechner S, Hoagland N, Hoekstra MF, Piwnica-Worms H, Cantley LC (1994). Use of an oriented peptide library to determine the optimal substrates of protein kinases. Curr Biol.

[B26] Maurer-Stroh S, Eisenhaber B, Eisenhaber F (2002). N-terminal N-myristoylation of proteins: refinement of the sequence motif and its taxon-specific differences. J Mol Biol.

[B27] Iakoucheva LM, Radivojac P, Brown CJ, O'Connor TR, Sikes JG, Obradovic Z, Dunker AK (2004). The importance of intrinsic disorder for protein phosphorylation. Nucleic Acids Res.

[B28] Tomii K, Kanehisa M (1996). Analysis of amino acid indices and mutation matrices for sequence comparison and structure prediction of proteins. Protein Eng.

[B29] Eisenberg D (1984). Three-dimensional structure of membrane and surface proteins. Annu Rev Biochem.

[B30] Vihinen M, Torkkila E, Riikonen P (1994). Accuracy of protein flexibility predictions. Proteins.

[B31] Feramisco JR, Glass DB, Krebs EG (1980). Optimal spatial requirements for the location of basic residues in peptide substrates for the cyclic AMP-dependent protein kinase. J Biol Chem.

[B32] Krigbaum WR, Komoriya A (1979). Local interactions as a structure determinant for protein molecules: II. Biochim Biophys Acta.

[B33] Nakashima H, Nishikawa K, Ooi T (1990). Distinct character in hydrophobicity of amino acid compositions of mitochondrial proteins. Proteins.

[B34] Geisow MJ, Roberts RDB (1980). Amino-Acid Preferences for Secondary Structure Vary with Protein Class. International Journal of Biological Macromolecules.

[B35] Kanehisa MI, Tsong TY (1980). Local hydrophobicity stabilizes secondary structures in proteins. Biopolymers.

[B36] Fasman GD (1976). Handbook of Biochemistry and Molecular Biology.

[B37] Kishimoto A, Nishiyama K, Nakanishi H, Uratsuji Y, Nomura H, Takeyama Y, Nishizuka Y (1985). Studies on the phosphorylation of myelin basic protein by protein kinase C and adenosine 3':5'-monophosphate-dependent protein kinase. J Biol Chem.

[B38] Ekdahl KN (1987). Rat liver fructose-1,6-bisphosphatase. Identification of serine 338 as a third major phosphorylation site for cyclic AMP-dependent protein kinase and activity changes associated with multisite phosphorylation in vitro. J Biol Chem.

[B39] Sewing A, Muller R (1994). Protein kinase A phosphorylates cyclin D1 at three distinct sites within the cyclin box and at the C-terminus. Oncogene.

[B40] NCBI FTP-site. ftp://ftp.ncbi.nlm.nih.gov/genomes/H_sapiens/protein/protein.fa.

[B41] Neuberger G, Kunze M, Eisenhaber F, Berger J, Hartig A, Brocard C (2004). Hidden localization motifs: naturally occurring peroxisomal targeting signals in non-peroxisomal proteins. Genome Biol.

[B42] Nielsen H, Brunak S, von Heijne G (1999). Machine learning approaches for the prediction of signal peptides and other protein sorting signals. Protein Eng.

[B43] Maurer-Stroh S, Gouda M, Novatchkova M, Schleiffer A, Schneider G, Sirota FL, Wildpaner M, Hayashi N, Eisenhaber F (2004). MYRbase: analysis of genome-wide glycine myristoylation enlarges the functional spectrum of eukaryotic myristoylated proteins. Genome Biol.

[B44] O'Connor E, Eisenhaber B, Dalley J, Wang T, Missen C, Bulleid N, Bishop PN, Trump D (2005). Species specific membrane anchoring of nyctalopin, a small leucine-rich repeat protein. Hum Mol Genet.

[B45] Dongen S (2005). Graph Clustering by Flow Simulation.

[B46] Schneider G, Neuberger G, Wildpaner M, Tian S, Berezovsky I, Eisenhaber F (2006). Application of a sensitive collection heuristic for very large protein families: evolutionary relationship between adipose triglyceride lipase (ATGL) and classic mammalian lipases. BMC Bioinformatics.

[B47] Ruvinsky I, Sharon N, Lerer T, Cohen H, Stolovich-Rain M, Nir T, Dor Y, Zisman P, Meyuhas O (2005). Ribosomal protein S6 phosphorylation is a determinant of cell size and glucose homeostasis. Genes Dev.

[B48] Ruvinsky I, Meyuhas O (2006). Ribosomal protein S6 phosphorylation: from protein synthesis to cell size. Trends Biochem Sci.

[B49] (2007). The pkaPS WWW-site:. http://mendel.imp.univie.ac.at/sat/pkaPS.

[B50] Eisenhaber B, Eisenhaber F, Maurer-Stroh S, Neuberger G (2004). Prediction of sequence signals for lipid post-translational modifications: insights from case studies. Proteomics.

[B51] Darius F, Rojas R (1994). "Simulated molecular evolution" or computer-generated artifacts?. Biophys J.

[B52] Bairoch A, Apweiler R, Wu CH, Barker WC, Boeckmann B, Ferro S, Gasteiger E, Huang H, Lopez R, Magrane M, Martin MJ, Natale DA, O'Donovan C, Redaschi N, Yeh LS (2005). The Universal Protein Resource (UniProt). Nucleic Acids Res.

[B53] Apweiler R, Bairoch A, Wu CH, Barker WC, Boeckmann B, Ferro S, Gasteiger E, Huang H, Lopez R, Magrane M, Martin MJ, Natale DA, O'Donovan C, Redaschi N, Yeh LS (2004). UniProt: the Universal Protein knowledgebase. Nucleic Acids Res.

[B54] Leinonen R, Diez FG, Binns D, Fleischmann W, Lopez R, Apweiler R (2004). UniProt archive. Bioinformatics.

[B55] (2007). Phospho.ELM: Database of S/T/Y phosphorylation sites. http://phospho.elm.eu.org/.

[B56] (2007). UNIPROT Protein Sequence Database. http://www.expasy.uniprot.org/.

[B57] Sherman F, Stewart JW, Tsunasawa S (1985). Methionine or not methionine at the beginning of a protein. Bioessays.

[B58] Moerschell RP, Hosokawa Y, Tsunasawa S, Sherman F (1990). The specificities of yeast methionine aminopeptidase and acetylation of amino-terminal methionine in vivo. Processing of altered iso-1-cytochromes c created by oligonucleotide transformation. J Biol Chem.

[B59] Swiderek K, Jaquet K, Meyer HE, Schachtele C, Hofmann F, Heilmeyer LM (1990). Sites phosphorylated in bovine cardiac troponin T and I. Characterization by 31P-NMR spectroscopy and phosphorylation by protein kinases. Eur J Biochem.

[B60] Mittmann K, Jaquet K, Heilmeyer LM (1990). A common motif of two adjacent phosphoserines in bovine, rabbit and human cardiac troponin I. FEBS Lett.

[B61] Kemp BE, Bylund DB, Huang TS, Krebs EG (1975). Substrate specificity of the cyclic AMP-dependent protein kinase. Proc Natl Acad Sci U S A.

[B62] Thorens B, Deriaz N, Bosco D, DeVos A, Pipeleers D, Schuit F, Meda P, Porret A (1996). Protein kinase A-dependent phosphorylation of GLUT2 in pancreatic beta cells. J Biol Chem.

[B63] Blind E, Bambino T, Huang ZM, Bliziotes M, Nissenson RA (1996). Phosphorylation of the cytoplasmic tail of the PTH/PTHrP receptor. J of Bone Miner Res.

[B64] Bylund DB, Krebs EG (1975). Effect of denaturation on the susceptibility of proteins to enzymic phosphorylation. J Biol Chem.

[B65] Lomako J, Whelan WJ (1988). The occurrence of serine phosphate in glycogenin: a possible regulatory site. Biofactors.

[B66] Dhillon AS, Pollock C, Steen H, Shaw PE, Mischak H, Kolch W (2002). Cyclic AMP-dependent kinase regulates Raf-1 kinase mainly by phosphorylation of serine 259. Mol Cell Biol.

[B67] James PH, Pruschy M, Vorherr TE, Penniston JT, Carafoli E (1989). Primary structure of the cAMP-dependent phosphorylation site of the plasma membrane calcium pump. Biochemistry.

[B68] Moss SJ, Doherty CA, Huganir RL (1992). Identification of the Camp-Dependent Protein-Kinase and Protein-Kinase-C Phosphorylation Sites Within the Major Intracellular Domains of the Beta-1-Subunit, Gamma-2S-Subunit, and Gamma-2L-Subunit of the Gamma-Aminobutyric-Acid Type-A Receptor. Journal of Biological Chemistry.

[B69] Shinyama H, Masuzaki H, Fang H, Flier JS (2003). Regulation of melanocortin-4 receptor signaling: agonist-mediated desensitization and internalization. Endocrinology.

[B70] Tullai JW, Cummins PM, Pabon A, Roberts JL, Lopingco MC, Shrimpton CN, Smith AI, Martignetti JA, Ferro ES, Glucksman MJ (2000). The neuropeptide processing enzyme EC 3.4.24.15 is modulated by protein kinase A phosphorylation. J Biol Chem.

[B71] Campbell DG, Hardie DG, Vulliet PR (1986). Identification of four phosphorylation sites in the N-terminal region of tyrosine hydroxylase. J Biol Chem.

[B72] Keutmann HT, Ratanabanangkoon K, Pierce MW, Kitzmann K, Ryan RJ (1983). Phosphorylation of human choriogonadotropin. Stoichiometry and sites of phosphate incorporation. J Biol Chem.

[B73] Henikoff S, Henikoff JG (1994). Position-based sequence weights. J Mol Biol.

[B74] Vingron M, Argos P (1989). A fast and sensitive multiple sequence alignment algorithm. Comput Appl Biosci.

[B75] Vingron M, Sibbald PR (1993). Weighting in sequence space: a comparison of methods in terms of generalized sequences. Proc Natl Acad Sci U S A.

[B76] Kendall M, Stuart A (1977). The Advanced Theory of Statistics.

[B77] Sunyaev SR, Eisenhaber F, Rodchenkov IV, Eisenhaber B, Tumanyan VG, Kuznetsov EN (1999). PSIC: profile extraction from sequence alignments with position-specific counts of independent observations. Protein Eng.

[B78] Hastie T, Tibshirani R, Friedman J (2001). The Elements of Statistical Learning: Data Mining, Inference and Prediction.

[B79] Eisenhaber B, Bork P, Eisenhaber F (2001). Post-translational GPI lipid anchor modification of proteins in kingdoms of life: analysis of protein sequence data from complete genomes. Protein Eng.

[B80] Eisenhaber F, Eisenhaber B, Maurer-Stroh S, Andrade MM (2003). Prediction of Post-translational modifications from amino acid sequence: Problems, pitfalls, methodological hints. Bioinformatics and Genomes: Current Perspectives.

[B81] Altschul SF, Boguski MS, Gish W, Wootton JC (1994). Issues in searching molecular sequence databases. Nat Genet.

[B82] (2007). XMGRACE Software Package. http://plasma-gate.weizmann.ac.il/Grace/.

[B83] Trafny EA, Xuong NH, Adams JA, Ten Eyck LF, Taylor SS, Sowadski JM, Madhusudan (1994). cAMP-dependent protein kinase: crystallographic insights into substrate recognition and phosphotransfer. Protein Sci.

[B84] Humphrey W, Dalke A, Schulten K (1996). VMD: visual molecular dynamics. J Mol Graph.

[B85] Zimmerman JM, Eliezer N, Simha R (1968). The characterization of amino acid sequences in proteins by statistical methods. J Theor Biol.

[B86] Notredame C, Higgins DG, Heringa J (2000). T-Coffee: A novel method for fast and accurate multiple sequence alignment. J Mol Biol.

[B87] Huang TS, Bylund DB, Stull JT, Krebs EG (1974). The amino acid sequences of the phosphorylated sites in troponin-I from rabbit skeletal muscle. FEBS Lett.

[B88] Li Y, van Kerkhof P, Marzolo MP, Strous GJ, Bu G (2001). Identification of a major cyclic AMP-dependent protein kinase A phosphorylation site within the cytoplasmic tail of the low-density lipoprotein receptor-related protein: implication for receptor-mediated endocytosis. Mol Cell Biol.

[B89] Wang LY, Taverna FA, Huang XP, MacDonald JF, Hampson DR (1993). Phosphorylation and modulation of a kainate receptor (GluR6) by cAMP-dependent protein kinase. Science.

[B90] Marchler-Bauer A, Anderson JB, DeWeese-Scott C, Fedorova ND, Geer LY, He S, Hurwitz DI, Jackson JD, Jacobs AR, Lanczycki CJ, Liebert CA, Liu C, Madej T, Marchler GH, Mazumder R, Nikolskaya AN, Panchenko AR, Rao BS, Shoemaker BA, Simonyan V, Song JS, Thiessen PA, Vasudevan S, Wang Y, Yamashita RA, Yin JJ, Bryant SH (2003). CDD: a curated Entrez database of conserved domain alignments. Nucleic Acids Res.

[B91] Bateman A, Coin L, Durbin R, Finn RD, Hollich V, Griffiths-Jones S, Khanna A, Marshall M, Moxon S, Sonnhammer EL, Studholme DJ, Yeats C, Eddy SR (2004). The Pfam protein families database. Nucleic Acids Res.

[B92] Harpaz Y, Gerstein M, Chothia C (1994). Volume changes on protein folding. Structure.

[B93] Karplus PA, Schulz GE (1985). Prediction of Chain Flexibility in Proteins - A Tool for the Selection of Peptide Antigens. Naturwissenschaften.

[B94] Cid H, Bunster M, Canales M, Gazitua F (1992). Hydrophobicity and structural classes in proteins. Protein Eng.

[B95] Chang CC, Lin CJ (2006). LIBSVM: a library for support vector machines. http://www.csie.ntu.edu.tw/~cjlin/libsvm/.

